# Identification of a Novel Lipoprotein Regulator of *Clostridium difficile* Spore Germination

**DOI:** 10.1371/journal.ppat.1005239

**Published:** 2015-10-23

**Authors:** Kelly A. Fimlaid, Owen Jensen, M. Lauren Donnelly, Michael B. Francis, Joseph A. Sorg, Aimee Shen

**Affiliations:** 1 Department of Microbiology and Molecular Genetics, University of Vermont, Burlington, Vermont, United States of America; 2 Program in Cellular, Molecular & Biomedical Sciences, University of Vermont, Burlington, Vermont, United States of America; 3 Department of Biology, Texas A&M University, College Station, Texas, United States of America; The University of Texas-Houston Medical School, UNITED STATES

## Abstract

*Clostridium difficile* is a Gram-positive spore-forming pathogen and a leading cause of nosocomial diarrhea. *C*. *difficile* infections are transmitted when ingested spores germinate in the gastrointestinal tract and transform into vegetative cells. Germination begins when the germinant receptor CspC detects bile salts in the gut. CspC is a subtilisin-like serine pseudoprotease that activates the related CspB serine protease through an unknown mechanism. Activated CspB cleaves the pro-SleC zymogen, which allows the activated SleC cortex hydrolase to degrade the protective cortex layer. While these regulators are essential for *C*. *difficile* spores to outgrow and form toxin-secreting vegetative cells, the mechanisms controlling their function have only been partially characterized. In this study, we identify the lipoprotein GerS as a novel regulator of *C*. *difficile* spore germination using targeted mutagenesis. A *gerS* mutant has a severe germination defect and fails to degrade cortex even though it processes SleC at wildtype levels. Using complementation analyses, we demonstrate that GerS secretion, but not lipidation, is necessary for GerS to activate SleC. Importantly, loss of GerS attenuates the virulence of *C*. *difficile* in a hamster model of infection. Since GerS appears to be conserved exclusively in related Peptostreptococcaeace family members, our results contribute to a growing body of work indicating that *C*. *difficile* has evolved distinct mechanisms for controlling the exit from dormancy relative to *B*. *subtilis* and other spore-forming organisms.

## Author Summary

*Clostridium difficile* is a spore-forming bacterium capable of causing severe diarrhea. The dormant spore-form of *C*. *difficile* is necessary to cause infection, since vegetative cells of this organism cannot survive in the presence of oxygen. Spores are difficult to eradicate because they can withstand extreme environmental conditions and chemical insults including antibiotics. However, since spores cannot grow, they must transform back into actively replicating cells once the appropriate environmental conditions are sensed through a process called germination. A key step during germination is the break-down of a specialized cell wall layer in the spore known as cortex by the SleC hydrolase. In this paper, we identify GerS as a novel lipid-modified protein that is important for *C*. *difficile* germination to occur. GerS is made at high levels during spore formation and gets packaged into mature spores. We show that GerS is required for the cortex hydrolase SleC to degrade the protective cortex layer, since a strain lacking GerS does not lose its cortex layer. Loss of GerS prevents *C*. *difficile* from causing infection in a hamster model of infection, suggesting that GerS is a novel target for drug development.

## Introduction

*Clostridium difficile* is a Gram-positive spore-former capable of causing diarrheal disease that can lead to fatal colitis. Disease symptoms are caused by the production of two toxins, TcdA and TcdB, which are secreted when *C*. *difficile* establishes infection in the gastrointestinal tract of mammals [1–3]. *C*. *difficile* infections have primarily been associated with individuals undergoing antibiotic therapy, but long hospitalizations, underlying comorbidities, community-acquired infections, and age-related risk factors have also been documented [4–6]. These complications lead to *C*. *difficile* disease treatment costs between $1–5 billion per year in the United States [7,8]. Of the 0.5 million *C*. *difficile* infections in the United States each year, approximately 30,000 lead to death [9]. These deaths are primarily due to recurrent *C*. *difficile* infections, which occur in ~20–30% of people that clear the first infection [9,10].

Since *C*. *difficile* is an obligate anaerobe, its endospore, or spore form, is responsible for initiating infection and mediating disease recurrence [11]. Spores are highly resistant, oxygen-tolerant, multi-layered structures composed of a tightly packed, dehydrated inner core surrounded by the inner forespore membrane, a germ cell wall, a thick modified peptidoglycan layer known as cortex, an outer forespore membrane, a series of proteinaceous layers known as the coat, and, in some spore formers, an outermost exosporium layer [12,13]. The specialized packaging of spores confers resistance to many chemical and physical insults and allows them to persist in the environment, and potentially an infected human, for long periods of time [1,14]. The dehydrated core renders spores metabolically dormant and is achieved by the displacement of water by calcium dipicolinic acid (Ca-DPA) in late stages of spore formation [15,16]. The thick cortex layer surrounding the core physically constrains its expansion and prevents hydration [17].

*C*. *difficile* infections begin when spores are ingested by a susceptible host and transit to the gastrointestinal (GI) tract [18–20]. In the GI tract, *C*. *difficile* spores sense specific bile salts, which induce them to transform into vegetative cells in a process known as germination [18,21]. While germination has been primarily characterized in the model organism *Bacillus subtilis* and in *C*. *perfringens* [13,22], recent studies in *C*. *difficile* have revealed that *C*. *difficile* uses a unique mechanism to regulate the initiation of spore germination [21,23–26].

While *B*. *subtilis* and *C*. *perfringens* employ highly conserved inner membrane germinant receptors to sense small molecule nutrients (germinants), which can be amino acids, sugars, and potassium ions [13], *C*. *difficile* and related Peptostreptococcaceae family members do not encode inner membrane germinant receptors [22,27]. Instead, *C*. *difficile* uses the subtilisin-like serine protease CspC as a germinant receptor [21] to sense bile salt germinants such as taurocholate [18,20,28–30]. Although *C*. *perfringens* encodes a CspC homolog and the related Csp family serine proteases, CspA and CspB [25,26], CspC is dispensable for germination in *C*. *perfringens* [25] in contrast with *C*. *difficile* [21]. Furthermore, *C*. *perfringens* CspC is catalytically competent and undergoes autoprocessing similar to other subtilisin-like serine proteases [26], whereas *C*. *difficile* CspC carries two mutations in its catalytic triad and lacks autoprocessing activity [21,23]. Unlike the catalytically competent *C*. *perfringens* CspA, *C*. *difficile* CspA is produced as a pseudoprotease that is fused to a catalytically competent CspB protease [23]. During spore formation, the *C*. *difficile* CspBA fusion protein undergoes interdomain processing, and the CspB domain is incorporated into mature spores [23].

Despite these differences, CspB in both *C*. *perfringens* and *C*. *difficile* functions to process the cortex lytic enzyme (CLE) SleC, which is found in dormant spores as the pro-SleC zymogen [21,23–26,31]. SleC degrades the cortex layer, which is essential for spore germination to proceed [32]. In the Clostridia, SleC targets the cortex-specific modification muramic-δ-lactam (MAL), which allows SleC to avoid degrading the germ cell wall of the outgrowing cell [33,34]. In *B*. *subtilis*, the cortex lytic enzymes CwlJ and SleB target MAL [16,35], although these enzymes exhibit little primary sequence homology to clostridial SleC. Cortex hydrolysis in *C*. *difficile* was recently shown to be required for Ca-DPA to be released from the core [36,37], whereas in *B*. *subtilis*, Ca-DPA is released before the cortex is hydrolyzed and actually activates CwlJ [38,39]. These observations indicate that different regulatory factors and mechanisms control germination in *C*. *difficile* relative to *B*. *subtilis* and even *C*. *perfringens*.

In this report, we describe the identification of a novel regulator of *C*. *difficile* spore germination, CD3464 in strain 630, herein referred to as GerS, which is conserved among sequenced Peptostreptococcaceae family members. Using a series of biochemical, genetic, and cell biological assays, we characterize the *gerS* ^−^ phenotype and identify the stage at which spore germination is arrested. We also demonstrate that GerS is essential for virulence in hamsters.

## Results

### Identification of GerS as a novel regulator of *C*. *difficile* spore germination

**Fig 1:**
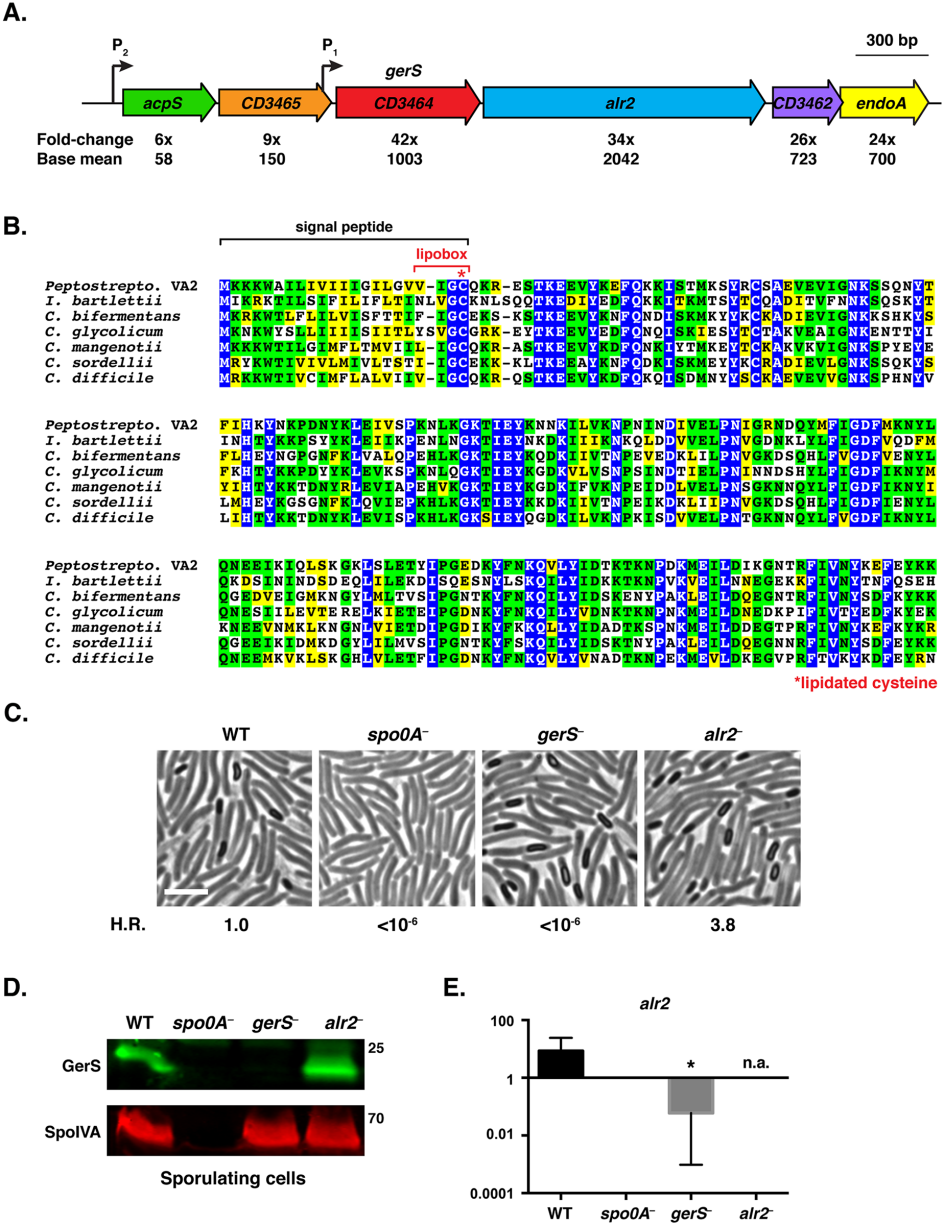
*C*. *difficile gerS* is highly induced during sporulation and encodes a protein important for heat-resistant spore formation. (A) Schematic of *C*. *difficile gerS* and *alr2* genomic loci. *gerS* and *alr2* are predicted to be part of an operon where transcription initiates from a P_1_ promoter immediately upstream of *gerS* (mapped by RNA-Seq transcriptional start site mapping, [42]) and potentially from the P_2_ promoter upstream of *acpS*. Fold-change represents the difference in gene expression between wild type and the *sigE* ^−^ mutant [41]. Base mean refers to the number of transcripts detected for the respective gene normalized to the length of that gene. (B) ClustalW alignment of GerS. Completely conserved, identical residues are blocked in blue, conserved identical residues are blocked in green, and conserved similar residues in yellow. The predicted signal peptide is bracketed in black, and the lipobox is bracketed in red. The lipobox cysteine predicted to be lipidated is designated by a red asterisk. The following sequences from Peptostreptococcaceae family members [27] were analyzed: *Peptostreptococcaceae* bacterium VA2 (WP_026902346), *Intestini bartlettii* (WP_007287647), *C*. *bifermentans* (WP_021430359), *C*. *glycolicum* (WP_018591922), *C*. *mangenotii* (WP_027700975), *C*. *sordellii* (CEK32529), and *C*. *difficile* (YP_001089984). (C) Phase contrast microscopy of the indicated *C*. *difficile* strains grown on sporulation media for 20 hrs. The *spo0A* ^−^ mutant cannot initiate sporulation [85]. The efficiency of heat-resistant spore formation (H.R.) was determined for each strain relative to wild type for three biological replicates. Scale bars represent 5 μm. (D) Western blot analysis of sporulating WT, *spo0A* ^−^, *gerS* ^−^, and *alr2* ^−^ cells. The mouse anti-SpoIVA antibody was used as a loading control [60]. (E) qRT-PCR analysis of *alr2* transcription in the indicated mutants. RNA was isolated from the indicated strains after sporulation was induced for 18 hrs. Transcript levels were normalized to the housekeeping gene *rpoB* using the standard curve method. Data represents the average of three biological replicates. Error bars indicate the standard error of the mean. n.a. indicates not applicable, since the region amplified spans the disrupted *alr2* gene. Statistical significance was determined using ANOVA and Tukey’s test (* p < 0.05).

We previously conducted RNA-Seq analyses of *C*. *difficile* sporulation-specific sigma factor mutants to identify gene products that might be required for spore formation and/or germination [40,41]. We hypothesized that highly expressed genes induced during sporulation would likely encode proteins that regulate spore formation and/or germination. *gerS* (*CD3464*) and *alr2* are the second and sixth most highly expressed, sporulation-induced genes [40,41], respectively, and their gene products have not been previously characterized. Interestingly, *alr2* is encoded downstream of *gerS* (Fig 1A), and these genes are part of a σ^E^-activated operon (S1 Fig, [42]). *alr2* encodes a putative alanine racemase that in *Bacillus*. spp. converts L-alanine to D-alanine and reduces the sensitivity of spores to L-alanine germinant [43–45]. *gerS* is predicted to encode a lipoprotein that appears to be unique to the Peptostreptococcaceae family (Fig 1B).

To test whether Alr2 or GerS regulate *C*. *difficile* sporulation and/or spore germination, we constructed TargeTron gene disruption mutants in *alr2* and *gerS* (S2 Fig). Analysis of the *alr2* and *gerS* mutants by phase contrast microscopy revealed that both strains produced phase-bright spores (Fig 1C). Fluorescence microscopy analyses indicated that *alr2* ^−^ and *gerS* ^−^ forespores appeared to develop similar to wild type (S3 Fig). However, when the *alr2* ^−^ and *gerS* ^−^ strains were tested for functional spore formation, the *gerS* mutant failed to produce detectable heat-resistant spores, while the *alr2* mutant produced wildtype levels of heat-resistant spores (Fig 1C). Western blot analysis confirmed that the *gerS* mutant was defective in producing GerS, while the *alr2* mutant produced wildtype levels of GerS (Fig 1D).

**Fig 2:**
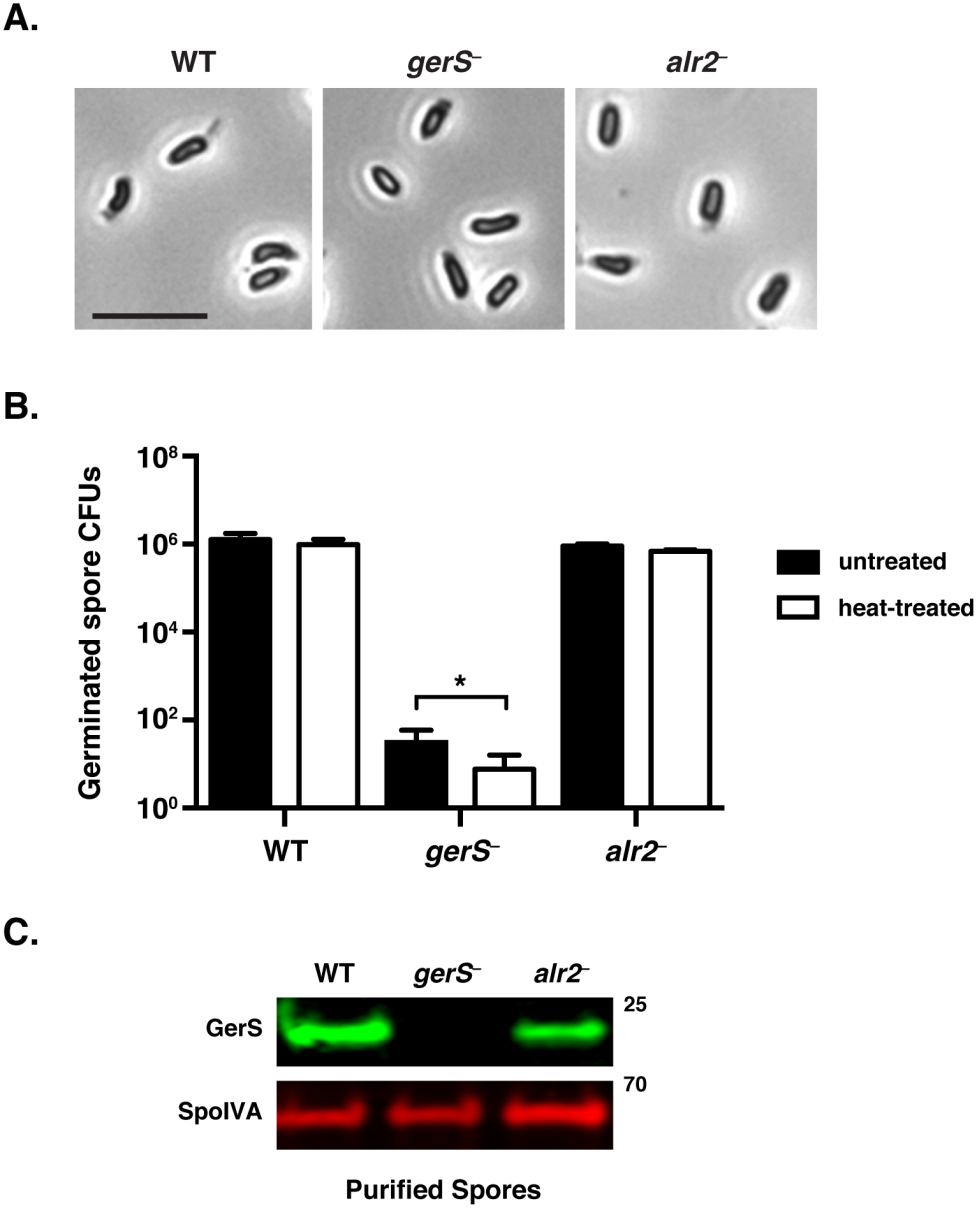
*C*. *difficile gerS* ^−^ mutant spores have a severe germination defect. (A) Phase contrast microscopy of *C*. *difficile* spores isolated from wildtype, *gerS* ^−^, and *alr2* ^−^ strains. *gerS* ^−^ and *alr2* ^−^ resemble wild type in size and their phase-bright appearance. Scale bars represent 5 μm. (B) Germination of wildtype, *gerS* ^−^, and *alr2* ^−^ spores following heat treatment. Heat-treated spores were incubated for 30 min at 60°C. Data represents the average of three biological replicates. Statistical significance was evaluated using ANOVA and Tukey’s test (* p < 0.05). (C) Western blot analysis of spores isolated from WT, *gerS* ^−^, and *alr2* ^−^ strains. Anti-SpoIVA was used as a loading control [60].

The inability of the *gerS* mutant to produce heat-resistant spores could be due to heat sensitivity [17,46] or a general defect in spore germination. To distinguish between these possibilities, we isolated spores from wild type and the *gerS* and *alr2* mutants and tested their ability to germinate following heat-treatment using a plate-based assay. No obvious defect in spore morphology was apparent when *gerS* ^−^ and *alr2* ^−^ spores were visualized by phase contrast microscopy (Fig 2A). However, *alr2* ^−^ spores germinated at wildtype levels, whereas *gerS* ^−^ spores exhibited an ~5-log defect in spore germination relative to wild type (Fig 2B). Heating wildtype and *alr2* ^−^ spores to 60°C for 30 min had no impact on spore germination, whereas heat treatment reduced the germination efficiency of *gerS* ^−^ spores by three-fold (p < 0.05, Fig 2B). Although a similar heat treatment potentiates *Bacillus* sp. spore germination [47–49], this effect has not been observed in *C*. *difficile* [37,50]. Western blot analyses verified that GerS is packaged into wildtype and *alr2* ^−^ mutant spores but not *gerS* ^−^ spores (Fig 2C). Taken together, these results strongly suggest that *gerS* ^−^ spores have a significant germination defect that is slightly heat sensitive. Furthermore, the germination defect of *gerS* mutant spores is unlikely to be caused by polar effects on *alr2* expression, since Alr2 itself is dispensable for heat-resistant spore formation.

### Complementation of the *gerS* mutant

To validate that the *gerS* mutant phenotype was due to absence of GerS, we complemented the mutant *in trans* by ectopically expressing *gerS* from its native promoter(s). Since *gerS* transcription originates from the proximal promoter (P_1_) directly upstream of *gerS* [42] and possibly the distal promoter upstream of *acpS* (P_2_, S4 Fig), we constructed *gerS* complementation constructs in which *gerS* transcription originates from the proximal promoter (P_1_, single) or from both P_1_ and P_2_ promoters (dual, including the two genes upstream of *gerS*). Heat resistance analyses revealed that the single and dual promoter complementation constructs both restored heat-resistant spore formation to wildtype levels (S4 Fig). Western blot analyses indicated that the dual and single promoter *gerS* complementation constructs restored GerS production to wildtype levels in the *gerS* ^−^ background (S4 Fig). We chose to use the dual promoter complementation construct, since it produced GerS levels that were most similar to wildtype carrying empty vector.

### GerS regulates cortex hydrolysis

We next sought to determine why *gerS* mutant spores exhibit such a strong germination defect. We first considered that GerS could affect the rate of spore germination, since a lipoprotein, GerD, controls the speed of germination in *B*. *subtilis* [51,52]. Loss of *B*. *subtilis* GerD results in an ~20-fold germination defect after a 15 hr incubation with germinant; however, after 48 hr, it resembles wild type [52]. Although *B*. *subtilis* GerD exhibits no homology to *C*. *difficile* GerS, we assessed whether *gerS* mutants germinated after prolonged incubation. After 48 hrs of germination on BHIS plates containing taurocholate, the change in number of colonies formed following *gerS* ^−^ spore germination was minimal and appeared to arise from spontaneous germination [53].

**Fig 3:**
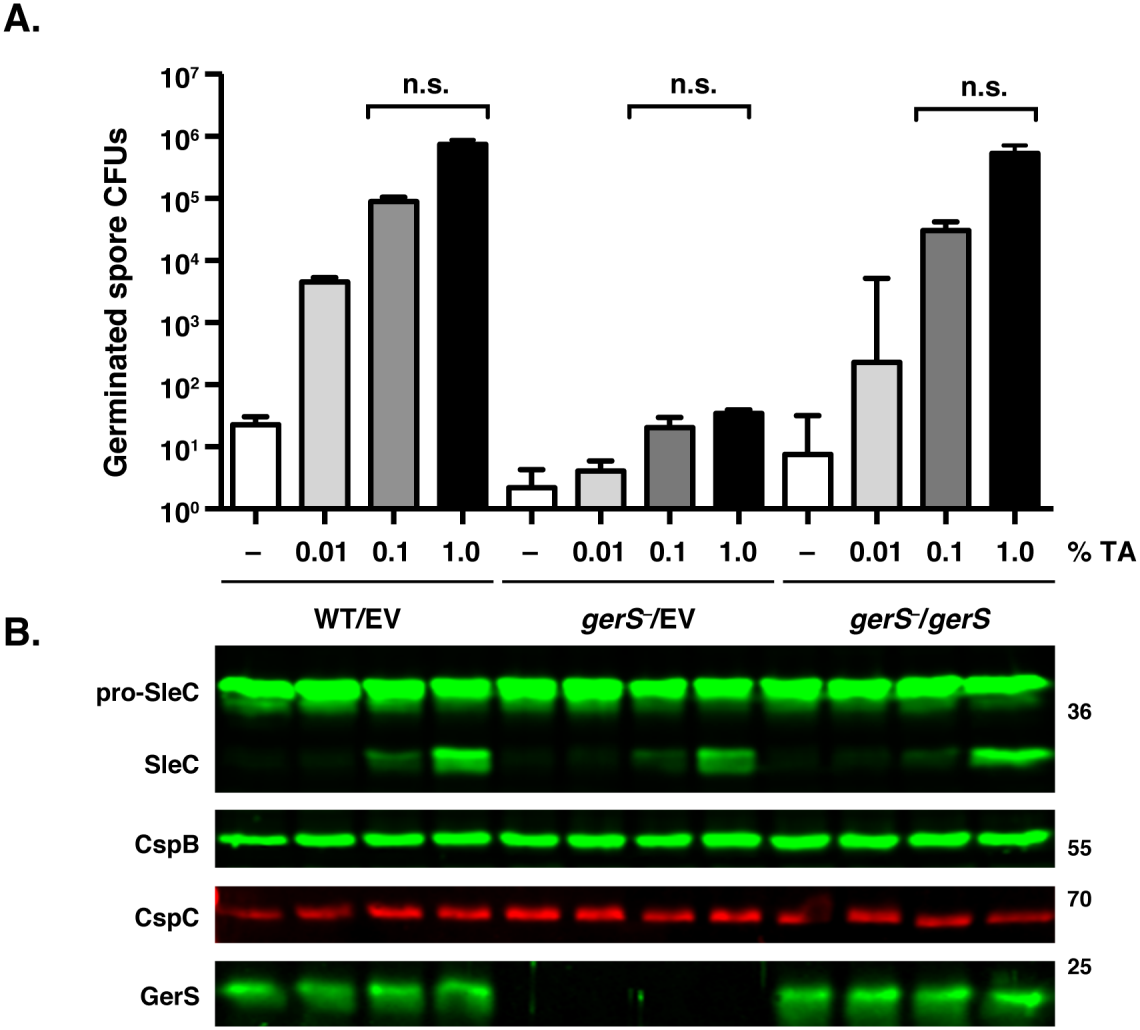
*gerS* ^−^ spores process pro-SleC in response to germinant. (A) *in vitro* germination of purified spores from wildtype carrying empty vector (WT/EV) and *gerS* ^−^ carrying empty vector (*gerS* ^−^/EV) or the *gerS* ^−^ complementation construct (*gerS* ^−^/*gerS*) spores in response to increasing amounts of taurocholate. Samples were plated on BHIS following taurocholate exposure. Data represents the average of three biological replicates. Statistical significance was evaluated using ANOVA and Tukey’s test. n.s. = no statistical significance. (B) Western blot analyses of samples from one representative replicate of the *in vitro* germination assay shown above. The zymogen pro-SleC is processed by CspB in response to taurocholate addition [23].

We next wondered whether GerS regulates germinant accessibility in *C*. *difficile* spores, since GerP in *B*. *subtilis* and *B*. *anthracis* facilitates the interaction of germinants to inner membrane germination receptors, potentially by altering coat permeability [54–56]. *Bacillus* spp. *gerP* mutants exhibit slower germination and require higher levels of germinant in order to achieve equivalent levels of germination as wild type. To test whether *C*. *difficile gerS* mutant spores are differentially sensitive to germinant, we compared the effect of increasing concentrations of taurocholate germinant on *gerS* ^−^ spores carrying empty vector (*gerS* ^−^/EV) relative to wildtype carrying empty vector (WT/EV) and *gerS* ^−^ spores carrying the wildtype complementation construct (*gerS* ^−^/*gerS*). *gerS* ^−^ spores exhibited a similar dose-dependent germination response to taurocholate as wild type and the *gerS* complementation spores, although *gerS* ^−^ spores still had an ~5-log defect in spore germination in the presence of 1% taurocholate (Fig 3A), which leads to germination levels equivalent to those obtained by plating on BHIS plates containing 0.1% taurocholate.

This result suggested that *gerS* ^−^ spores can sense germinant similar to wildtype spores. Consistent with this finding, no difference in the levels of CspC germinant receptor and CspB germination protease were observed between the strains by Western blotting (Fig 3B), and no difference in CspB-mediated processing of SleC in response to increasing amounts of germinant was observed. Since CspB-mediated processing of *C*. *perfringens* SleC activates its cortex hydrolase function [26], and CspB-mediated processing of *C*. *difficile* SleC is required for optimal spore germination [23], these results suggested that GerS acts after SleC-mediated cortex hydrolysis.

**Fig 4:**
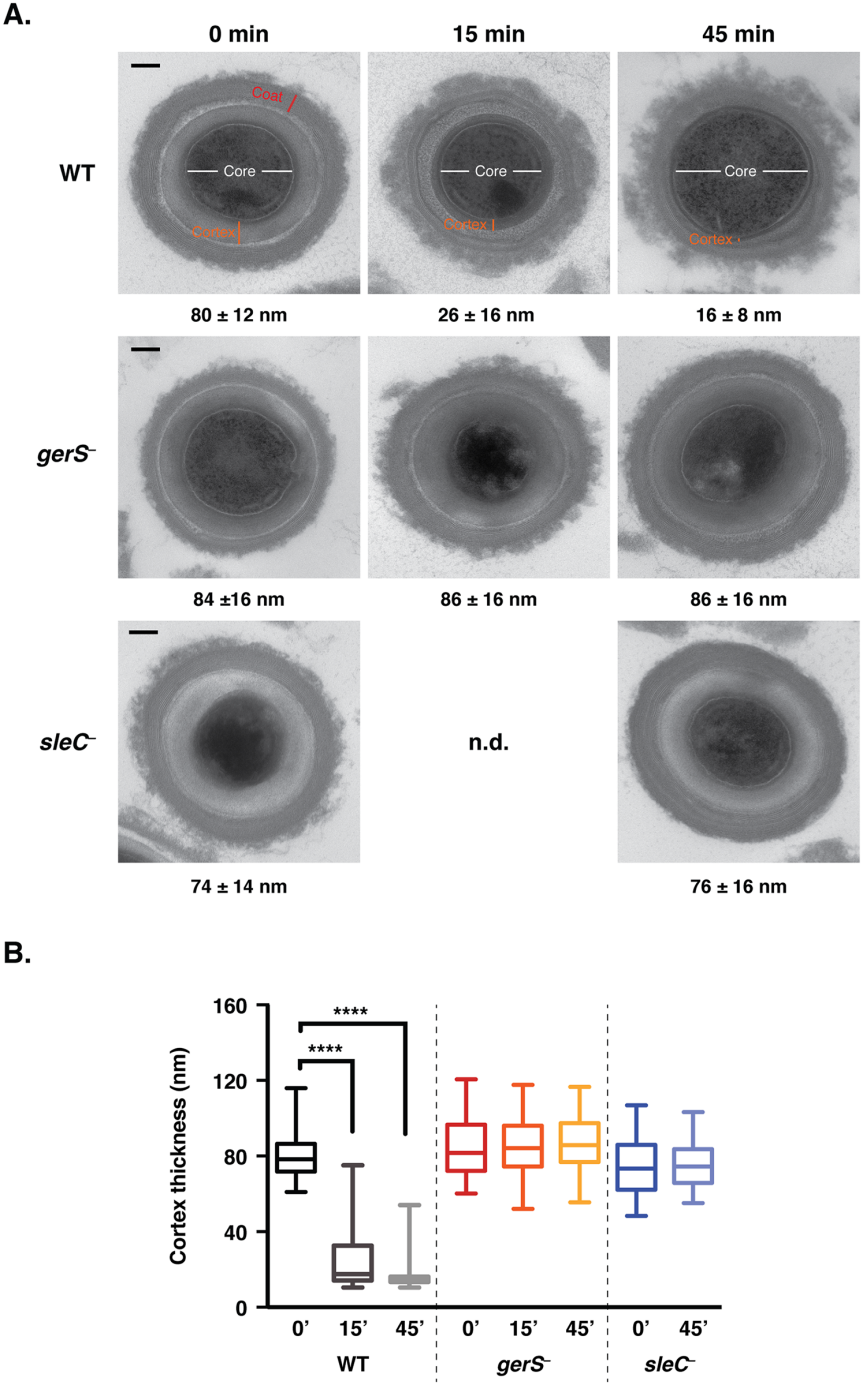
*gerS* ^−^ spores are defective in cortex hydrolysis. (A) TEM analyses of wildtype, *gerS* ^−^ and *sleC* ^−^ spores at time 0’, 15’, and 45’ post exposure to taurocholate. The 0’ timepoint was taken before taurocholate was added. *sleC* ^−^ spores were used as a negative control, since they do not undergo cortex hydrolysis [36,37]. The average cortex thickness was determined for each sample from a minimum of 50 spores at each timepoint. n.d. designates not determined. Representative images are shown. Scale bars designate 100 nm. (B) Box and whiskers plot of cortex thickness of the *in vitro* germination assay shown in (A). Statistical significance was determined using ANOVA and Tukey’s test (**** p < 0.0001).

In order to test this hypothesis, we developed a transmission electron microscopy (TEM) assay to visualize and quantify cortex hydrolysis. Although cortex hydrolysis can be measured biochemically [24,36,57], it is difficult to obtain the amount of spores required for these analyses using the JIR8094 strain background. For the TEM assay, wildtype, *gerS* ^−^, and *sleC* ^−^ spores were exposed to germinant for 45 min, and cortex thickness was measured over time for a minimum of 50 spores per time point (Fig 4). Within 15 min of exposure to germinant, cortex thinning was visible in wildtype spores (Fig 4A), and the average thickness decreased by 3-fold (p < 0.0001, Fig 4B). Cortex thickness decreased even further at 45 min. In contrast with wild type, no change in cortex thickness was observed in either *sleC* ^−^ or *gerS* ^−^ spores even after 45 min of incubation with germinant (Fig 4B). Thus, even though taurocholate induces CspB-mediated pro-peptide removal from the pro-SleC zymogen in *gerS* ^−^ spores (Fig 3), SleC does not appear to be active (Fig 4). These results suggest that GerS may regulate SleC activity through an unknown post-translational mechanism or by altering the availability of the SleC substrate, MAL, in the cortex layer.

**Fig 5:**
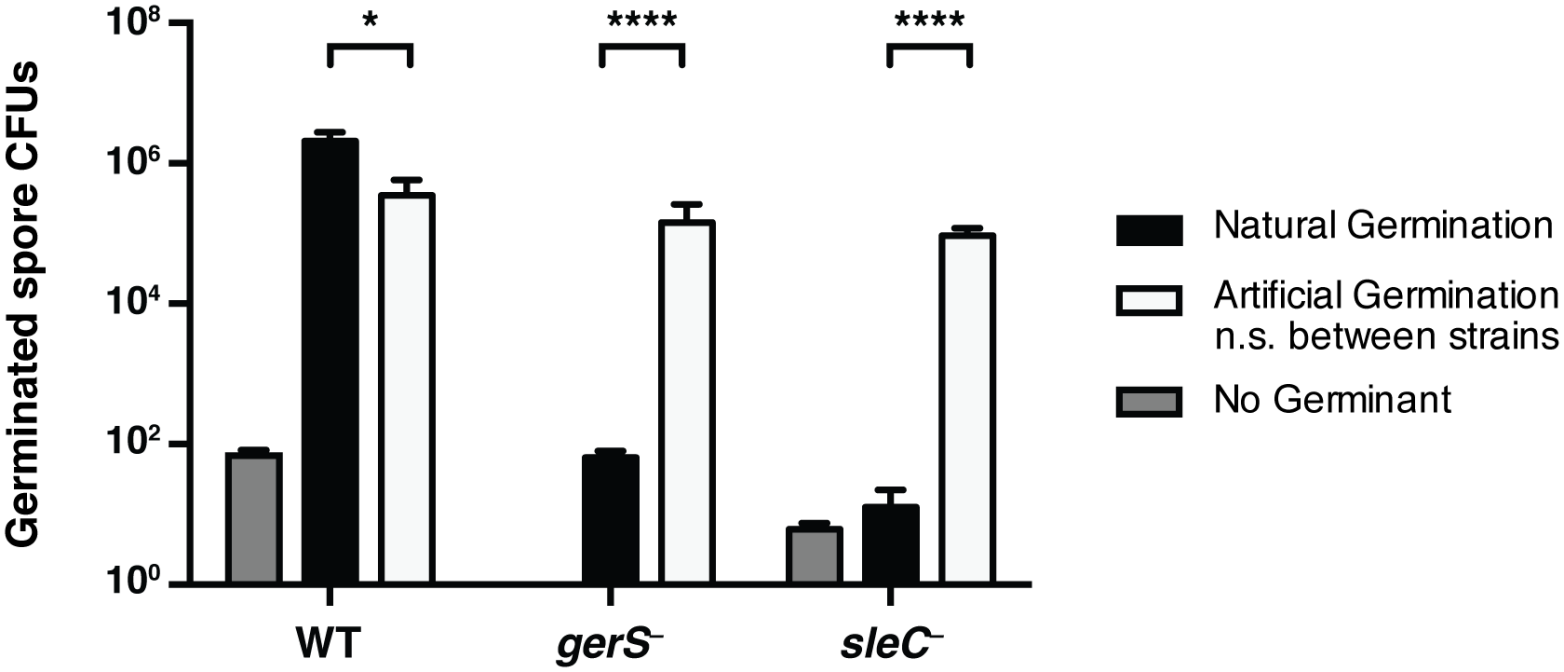
Artificial germination of *gerS* ^−^ bypasses its germination defect. Wildtype, *gerS* ^−^, and *sleC* ^−^ spores were incubated with no germinant, taurocholate (natural germination) or thioglycollate and lysozyme (artificial germination). Mutants defective in cortex hydrolysis can be artificially germinated [58]. Data represents the average of 4 biological replicates. No statistical significance was observed between strains subjected to artificial germination, in contrast with natural germination. Statistical significance was determined using ANOVA and Tukey’s test (* p < 0.05; **** p < 0.001).

If SleC activity is indeed dependent on the presence of GerS, it should be possible to bypass the need for SleC-mediated cortex hydrolysis by artificially germinating *gerS* ^−^ spores. During artificial germination, a reducing agent, thioglycollate, is added to permeabilize the coat layers followed by lysozyme addition to degrade the cortex layer [13,58]. Treatment of wildtype, *gerS* ^−^, and *sleC* ^−^ spores with thioglycollate and lysozyme restored outgrowth to *gerS* ^−^ and *sleC* ^−^ spores (Fig 5); no statistically significant difference in artificial germination between wildtype, *gerS* ^−^, *sleC* ^−^ spores was observed. In contrast, wildtype spores germinated much more efficiently than the mutants upon “natural” exposure to taurocholate. The small amount of germination observed in *sleC* ^−^ spores is likely due to spontaneous germination [37,46], which can occur even in the absence of the germinant receptor [21].

### Ca-DPA release does not occur in the absence of GerS

Since recent studies have shown that Ca-DPA release immediately follows cortex hydrolysis [36,37], we next tested whether the *gerS* mutant releases Ca-DPA in response to germinant (S5 Fig). Whereas wildtype spores released ~80% of their Ca-DPA stores in response to germinant, *gerS* ^−^ spores released <5% of their Ca-DPA stores. Since wildtype and *gerS* ^−^ spores contained similar amounts of Ca-DPA (88%, S5 Fig), Ca-DPA storage does not appear to be affected by the *gerS* mutation, consistent with the recent observation that Ca-DPA release depends on cortex hydrolysis in *C*. *difficile* [36,37] in contrast with *B*. *subtilis* [13,38].

### GerS localizes to a “coat-extractable” (CE) fraction

**Fig 6:**
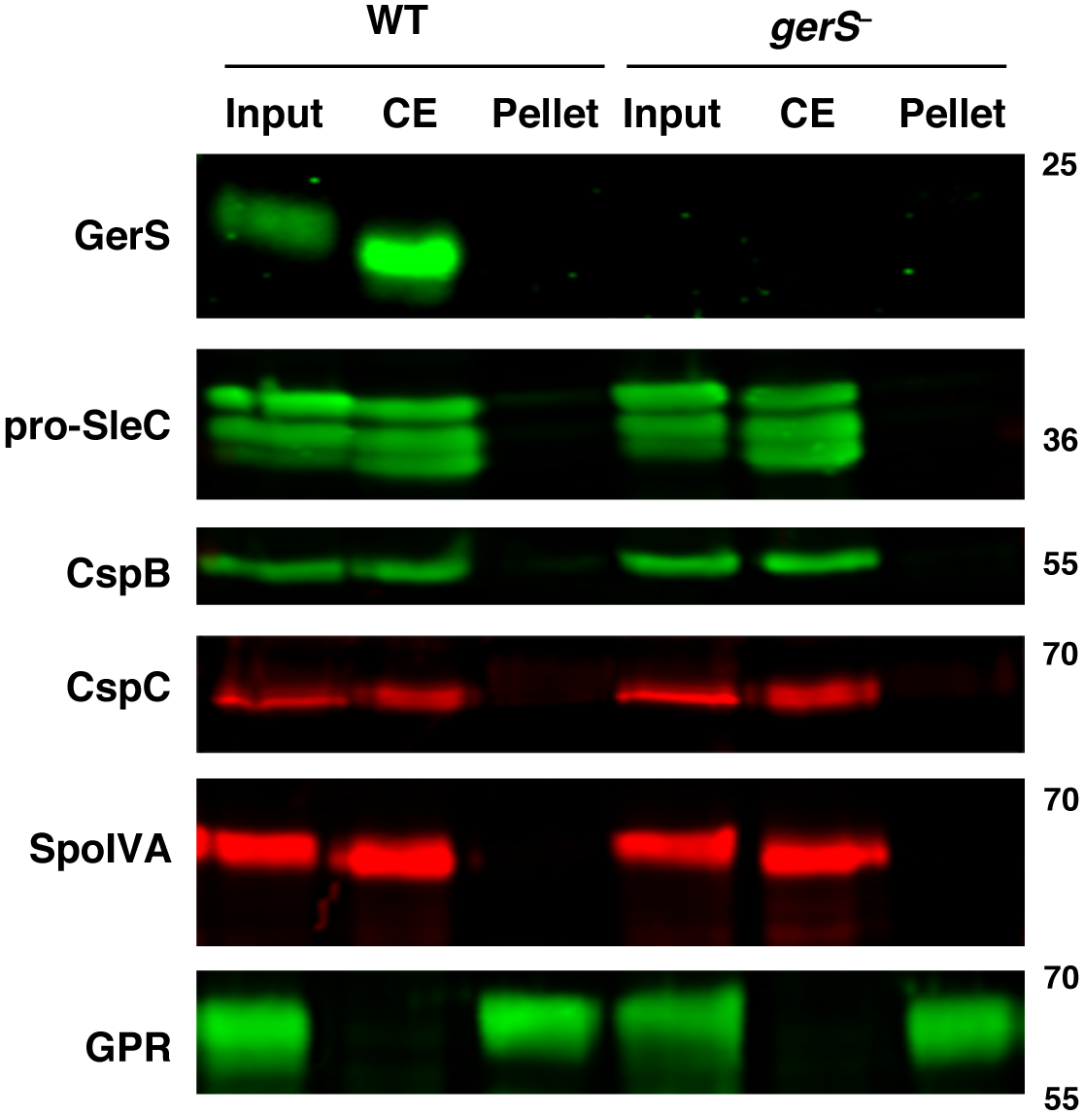
Germination proteins localize to a “coat-extractable” (CE) fraction. Western blot analyses of “coat-extractable” (CE) and decoated spore lysate (pellet) fractions from wildtype and *gerS* ^−^ spores. It should be noted that the CE fraction likely includes proteins localized to the cortex and outer-forespore membrane. Input represents the whole spore lysate without fractionation. SpoIVA is a coat morphogenetic protein [60]. GPR (germination protease) is localized to the core of spores [40,61,62].

Having shown that GerS is important for SleC activity, we next wanted to understand how GerS carries out its function. We first tested whether GerS and SleC were present in the “coat-extractable” (CE) fraction. To this end, we subjected wildtype and *gerS* ^−^ mutant spores to a mild boric acid decoating treatment [57] to generate a CE supernatant fraction and a pellet fraction. Western blotting of these fractions revealed that both SleC and GerS co-localized to the CE fraction in wildtype spores but not to the pellet fraction, which consists of decoated spore lysate (Fig 6). Analysis of germination regulators CspC and CspB revealed that they also are concentrated in the CE fraction of wildtype and *gerS* ^−^ spores. These results indicate that the germination regulators GerS, SleC, CspB, and CspC are located in a similar cellular fraction. Since *C*. *perfringens* SleC has been shown to localize to the cortex using immunoelectron microscopy [59], and CspB and SleC were recently reported to localize to a CE fraction in *C*. *perfringens* [31], these results imply that the CE fraction includes cortex and outer forespore membrane proteins in *C*. *perfringens* and likely in *C*. *difficile* (although it remains formally possible that SleC does not localize to the cortex region in *C*. *difficile*). Importantly, the coat morphogenetic protein SpoIVA [60] localized exclusively to the CE fraction of wildtype and *gerS* ^−^ mutant spores, whereas the forespore-localized germination protease (GPR) [40,61,62] was found exclusively in the pellet fraction of these spores.

### Secretion, but not lipidation, of GerS is required for its function

**Fig 7:**
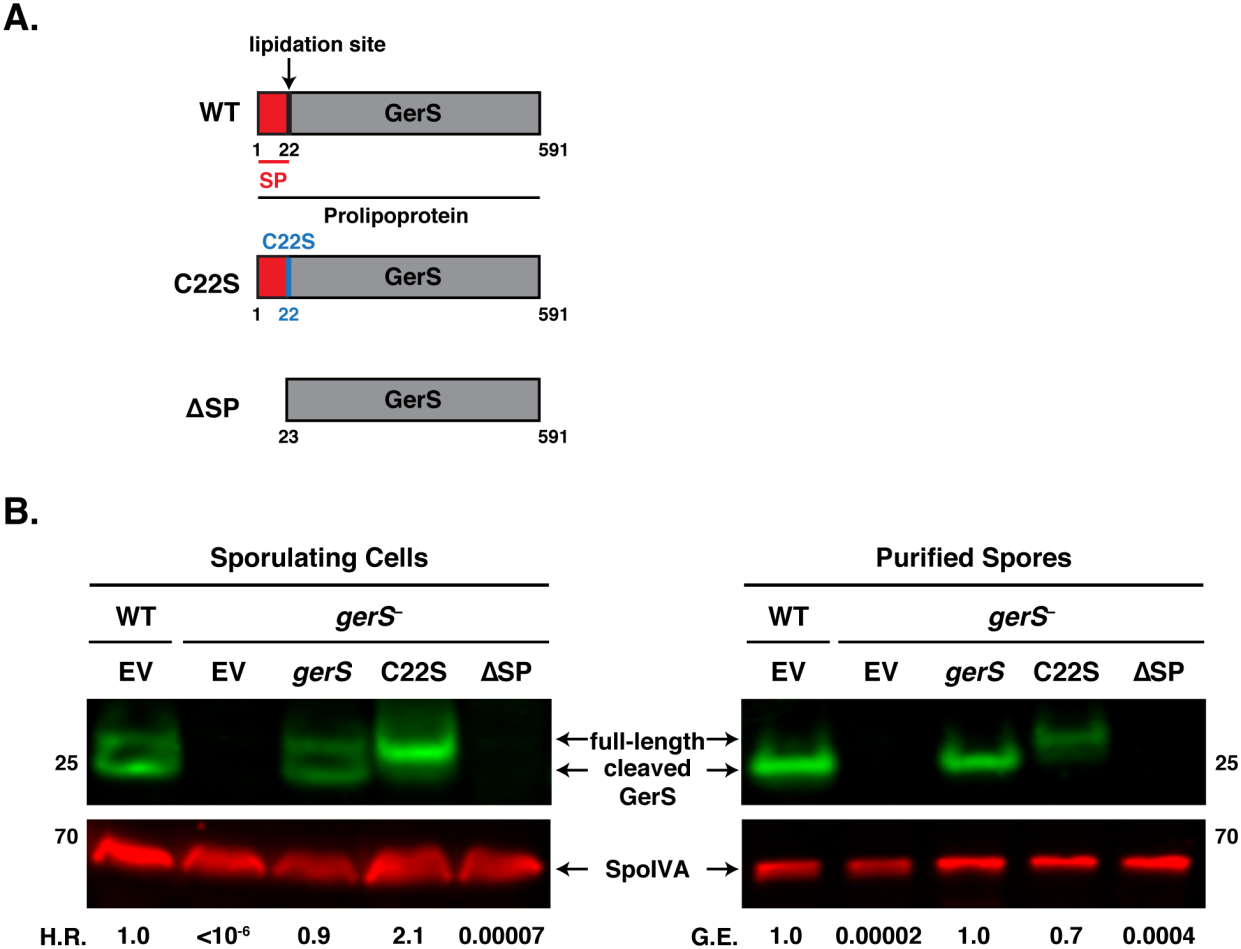
The signal peptide of GerS, but not its lipidation site, is required for germination. (A) Schematic of *gerS* complementation constructs. C22S designates a construct encoding a mutation of the invariant lipidation site cysteine to serine. ΔSP designates a construct encoding a truncated GerS lacking its signal peptide. (B) Western blot analyses of GerS in sporulating cells and purified spores from wild type carrying empty vector (EV) and the *gerS* mutant carrying either empty vector or the indicated complementation constructs. SpoIVA was used as a loading control [60]. The efficiency of heat-resistant spore formation (H.R.) during sporulation is shown, as is the germination efficiency (G.E.) of purified spores. Data is representative of at least 4 replicates.

Since GerS is predicted to be a lipoprotein based on the presence of a putative N-terminal signal peptide containing a lipobox [63–65], we tested whether GerS undergoes lipidation and whether its function depends on lipidation and/or secretion. The signal peptide of lipoproteins directs their transport across membranes after which Lgt, a prolipoprotein diacylglyerol transferase, adds a diacylglycerol group to the lipobox cysteine via a thioether bond. Following lipidation, the lipoprotein signal peptidase (Lsp) cleaves off the signal peptide, and the lipoprotein inserts into the plasma membrane in Gram-positive bacteria [63]. Since mutation of the conserved cysteine residue in the lipobox to serine is sufficient to prevent lipidation [63–65], we complemented the *gerS* mutant with a construct that produces GerS carrying a cysteine 22 to serine (C22S) mutation. We also complemented the *gerS* mutant with a construct that deletes the GerS signal peptide sequence to prevent secretion (ΔSP, Figs 1B and 7A). The C22S complementation strain produced heat-resistant spores at levels comparable to wild type, whereas the ΔSP complementation strain exhibited a >4-log decrease in functional (heat-resistant) spore formation relative to wildtype (H.R., Fig 7B). These results suggest that secretion but not lipidation is required for GerS to activate cortex hydrolysis. Western blot analyses of the complementation strains revealed that only full-length GerS was detectable in the C22S strain, whereas both full-length and cleaved GerS were observed in wild type and the wildtype *gerS* complementation strain. These observations strongly suggest that Cysteine 22 is important for cleavage of the signal peptide, similar to other lipoproteins [63–65]. Neither full-length nor cleaved GerS could be detected in ΔSP sporulating cells, implying that loss of the signal peptide leads to destabilization of GerS (Fig 7B). To test this hypothesis, we measured *gerS* transcript levels in the complementation strains by qRT-PCR relative to wildtype carrying empty vector. The *gerS* ^−^ complementation strains all produced an excess of *gerS* transcripts relative to wildtype carrying empty vector; this over-expression is likely due to the multi-copy nature of the pMTL83151 plasmid used for complementation (S6 Fig). Thus, GerS lacking its signal peptide appears to be unstable in the mother cell cytosol of sporulating cells.

Consistent with our analyses of sporulating cells, purified spores from the C22S strain germinated at wildtype levels, while ΔSP spores exhibited an ~4-log defect in germination relative to wild type (Fig 7B). Only full-length GerS was detected in C22S spores, whereas only cleaved GerS was detected in wildtype spores carrying empty vector and *gerS* ^−^ spores carrying the wildtype complementation construct. GerS was undetectable in ΔSP spores. Taken together, these analyses suggest that GerS secretion across the mother cell-derived membrane is necessary for GerS function, while lipidation and signal peptide removal are dispensable for GerS to activate cortex hydrolysis.

To test whether alterations to the signal peptide affected the heat sensitivity of *gerS* ^−^ mutant spores, we heated spores for 30 min at 60°C prior to plating on media containing taurocholate germinant. As expected, heat treatment had no impact on the germination of wildtype spores carrying empty vector or wildtype complementation spores (S7 Fig). No difference in spore germination between untreated and heat-treated C22S or ΔSP spores was observed. In contrast, *gerS* ^−^ mutant spores carrying empty vector showed a statistically significant decrease in the number of germinating spores following heat treatment (p < 0.01), similar to results with *gerS* ^−^ spores (Fig 2B).

### GerS is necessary to cause disease in hamsters

**Fig 8:**
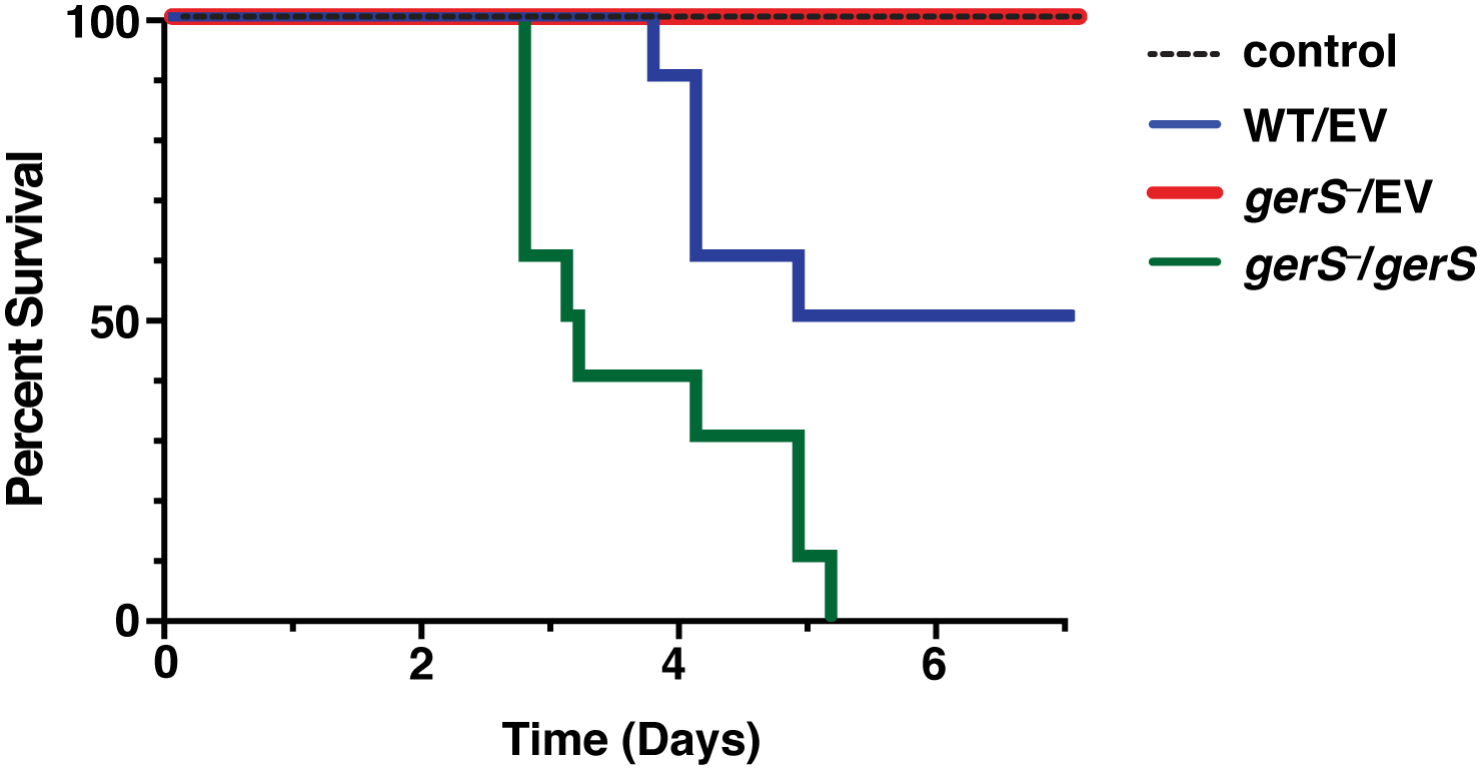
GerS is required for virulence. Kaplan-Meier survival curve of clindamycin-treated Syrian hamsters inoculated with 1,000 isolated spores of wildtype carrying empty vector (WT/EV), the *gerS* mutant carrying either empty vector (*gerS* ^−^/EV), or the wildtype complementation construct (*gerS* ^−^/*gerS*). The control designates antibiotic-treated hamsters that were not inoculated with spores.

Since bile acid-mediated germination has previously been shown to be important for *C*. *difficile* pathogenesis [21], we tested whether *gerS* ^−^ could cause disease in the hamster model of *C*. *difficile* infection (CDI). Hamsters inoculated with *gerS* ^−^ spores carrying empty vector had a 100% survival 7 days post inoculation, whereas wildtype spores carrying empty vector resulted in 50% survival at the same time point (Fig 8). Inoculation with the *gerS* ^−^/*gerS* construct resulted in 100% of the hamsters being euthanized by day 5 after inoculation. These results indicate that the *gerS* mutant’s *in vitro* germination defect correlates with an inability to cause disease in a hamster model of CDI.

Since a possible explanation for the greater lethality of the *gerS* ^−^/*gerS* strain might be a faster rate of spore germination relative to wildtype spores carrying empty vector, we analyzed the rate of germination initiation by measuring the decrease in optical density at 600 nm when spores form the complementation strains were exposed to taurocholate germinant (S8 Fig). This assay revealed that the C22S and *gerS* ^−^/*gerS* strains germinated with similar kinetics as wild type, albeit slightly less efficiently, whereas no major change in OD_600_ was observed for ΔSP and *gerS* ^−^/EV spores, as expected.

## Discussion

Recent studies of *C*. *difficile* spore germination have uncovered a unique signaling pathway for sensing bile salt germinants and initiating spore outgrowth relative to previously studied organisms [12]. Although the germination regulators SleC and the Csp family proteases are conserved between *C*. *difficile* and *C*. *perfringens* [22], they can have different functions and/or activities in these organisms [21,23–26]. In this study, we identified a novel protein specific to *C*. *difficile* and related Peptostreptococcaceae family members that functions as a critical regulator of SleC cortex hydrolase activity and is essential for germination *in vivo* in a hamster model of infection under the conditions tested. While *C*. *difficile* strain JIR8094 contains mutations in the flagellar operon that impacts motility and toxin gene expression, a *gerS* mutant nevertheless causes significantly less disease than wild type JIR8094.

In particular, we showed that GerS regulates SleC activity downstream of CspB-mediated processing of SleC. This processing event had previously been thought to be sufficient to activate SleC’s cortex hydrolase activity, since studies in *C*. *perfringens* showed that CspB-mediated cleavage of the pro-SleC zymogen was necessary for SleC to degrade cortex fragments *in vitro* [26,57], and loss of *C*. *difficile* CspB protease activity markedly reduced SleC processing and spore germination [23]. However, unlike *C*. *perfringens* SleC, full-length *C*. *difficile* SleC can degrade cortex fragments *in vitro* [33], calling into question why SleC does not automatically degrade cortex in dormant spores. It will be important in future studies to precisely determine the impact of pro-peptide removal in activating SleC function *in vitro* and in *C*. *difficile*.

**Fig 9:**
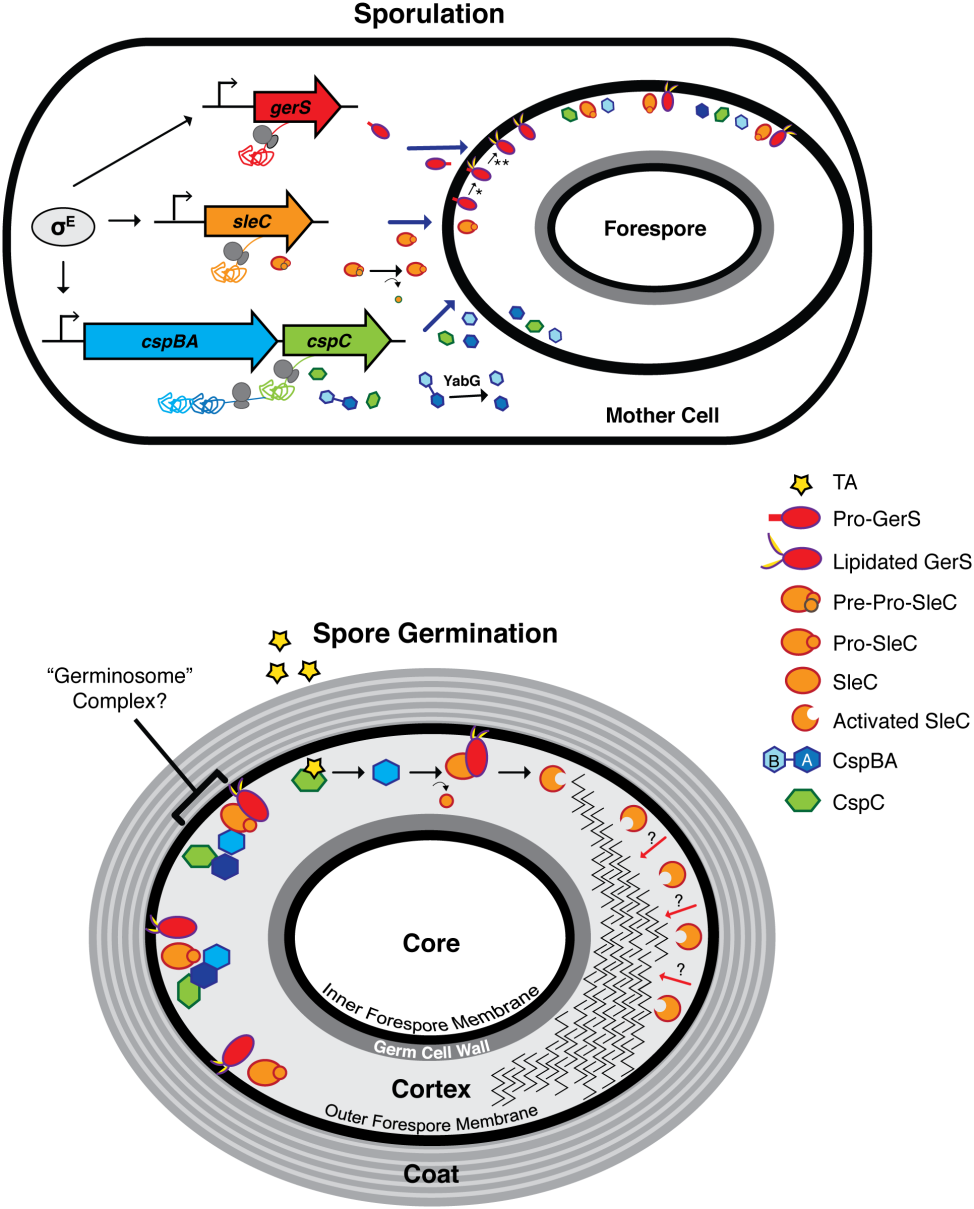
Model of *C*. *difficile* germination regulator production and localization during sporulation and germination. The sporulating cell depicts transcription of germination regulator genes and translation of the resulting transcripts. *gerS*, *sleC*, and *cspBAC* are all controlled by σ^E^ in the mother cell. σ^G^ may regulate the expression of gene products involved in germination, but these are unknown. In the mother cell, removal of the pre-domain is depicted by the curved arrow, while interdomain cleavage of CspBA is demarcated by the red X; these processing events occur during spore maturation [23]. Solid blue arrows designate transport of proteins from the mother cell into the forespore compartment. In mature spores, the germination regulators, GerS, SleC, CspC, and CspB, likely localize to the cortex region and/or to the outer forespore membrane. The possible association of these regulators into a “germinosome” complex [86] is designated by the bracket. The taurocholate (TA) germinant is depicted by the red star. Red arrows depict SleC-mediated cortex hydrolysis from the outer forespore membrane to the inner forespore membrane. * lipidation event; ** signal peptide cleavage event; dashed arrow designates an unknown event.

How then does GerS regulate SleC activity? Our results indicate that *gerS* is under the control of the mother cell-specific sigma factor σ^E^ (Fig 1A) and thus should be produced in the mother cell cytosol [40,41,61]. Deletion of the signal peptide from GerS destabilizes GerS in sporulating cells (Fig 7 and S6 Fig). This observation is consistent with the notion that GerS is transported across the outer forespore membrane into the cortex region during sporulation (Fig 9); more evidence is nevertheless needed to test this hypothesis. Since mutation of the invariant cysteine in the GerS lipobox prevents signal peptide removal but does not affect GerS function (Fig 7), the signal peptide of GerS C22S may insert into the outer forespore membrane where it can apparently function like lipidated wildtype GerS (Fig 7). Although mutation of the invariant lipobox cysteine frequently disrupts lipoprotein function [51,66,67], lipidation of some bacterial lipoproteins can be dispensable for their activity because they remain embedded in the plasma membrane through their signal peptide [63,68]. These observations suggest that GerS may exert its function on the surface of the outer forespore membrane facing the cortex (Fig 9). Notably, SleC activity also appears to be localized to this region, since TEM analyses of germinating wildtype spores revealed that cortex thinning initiates at the outer forespore membrane and radiates inward in *C*. *difficile* (Fig 4). While more studies are clearly needed to determine the exact locations of SleC and GerS in mature spores, our results suggest that these germination regulators may be localized to the outer forespore membrane, which likely fractionates with the coat (Fig 9), raising the intriguing possibility that GerS retains SleC at this site. It will be interesting to determine in future work whether GerS acts as a direct or indirect activator of SleC and/or whether GerS is necessary for SleC to recognize its cortex substrate, for example by controlling the predicted modification of NAM residues to muramic acid δ-lactam in the cortex [34,35], particularly since GerS lacks homology to other proteins aside from its lipobox.

Although GerS carries a signal peptide that directs its secretion across mother cell-derived membranes (Fig 9), SleC lacks a canonical N-terminal signal sequence. Thus, it is unclear how SleC is transported across the outer forespore membrane so that it can bind its cortex substrate. Similarly, how CspB is transported across this mother cell-derived membrane to cleave pro-SleC, and how CspC is presumably translocated across this membrane to activate CspB, remains unknown, since both CspB and CspC lack a canonical signal sequence.

Intriguingly, all the known germinant regulators in *C*. *difficile*, CspC, CspBA, SleC, and GerS, are produced under the control of mother cell-specific sigma factors [40,42,61]. In contrast, the germination regulators of *B*. *subtilis*, the GerAA-AC complex and GerD, are all under the control of the forespore-specific sigma factor σ^G^ [69]. These observations suggest that the topology of germination signaling differs significantly between *C*. *difficile* and *B*. *subtilis*. In *B*. *subtilis*, the germinant receptors are located in the inner forespore membrane [70–72], since decoated spores germinate efficiently [73]. Germinant sensing stimulates release of Ca-DPA from the core through the inner forespore membrane-localized channel SpoVAC [13]; Ca-DPA then activates the CwlJ cortex lytic enzyme [46]. In *C*. *difficile*, the germinant receptor CspC, the germination protease CspB, the cortex hydrolase SleC, and the lipoprotein GerS, all localize to the CE fraction (Fig 5). Thus, these regulators are unlikely to be associated with the inner forespore membrane in contrast with *B*. *subtilis*. Since SleC cortex hydrolase activation precedes Ca-DPA release in *C*. *difficile* (S5 Fig, [21,37]), the germinant signal appears to travel from the outside-in, whereas in *B*. *subtilis* the signal appears to travel from the inside-out.

While our genetic analyses demonstrated that GerS is a key germination regulator in *C*. *difficile*, they also showed that Alr2, a putative alanine racemase, is dispensable for germination (Fig 2). It should be noted that this observation does not exclude the possibility that Alr2 could alter the sensitivity of *C*. *difficile* spores to L-alanine, which has been shown to function as a co-germinant for *C*. *difficle in vitro* [74]. In *B*. *anthracis* and *B*. *cereus*, the Alr2 homolog alanine racemase converts L-alanine, a known germinant, to D-alanine to reduce the sensitivity of spores to L-alanine germinant [43,44,75]. Whether Alr2 modulates *C*. *difficile* spore germination remains to be determined, in particular whether it functions in suppressing germination. However, Howerton and Abel-Santos have shown that D-alanine is not an inhibitor of *C*. *difficile* spore germination [74], suggesting that Alr2 plays little role in *C*. *difficile* spore germination or has an as-yet-unknown function.

In summary, in identifying a novel germination regulator conserved in *C*. *difficile* and other Peptostreptococcaceae family members, our study reveals yet another difference between the regulation of spore germination in *C*. *difficile* relative to *B*. *subtilis* and *C*. *perfringens*. While many unanswered questions remain, cortex hydrolysis in *C*. *difficile* appears to be subject to an additional level of regulation during germination by GerS. Thus, GerS could be a potential target for inhibiting *C*. *difficile* disease transmission, especially given its limited conservation in spore-forming organisms.

## Materials and Methods

### Bacterial strains and growth conditions

*C*. *difficile* strains are listed in Table 1 and derive from the parent strain JIR8094, an erythromycin-sensitive derivative of the sequenced clinical isolate 630. *C*. *difficile* strains were grown on solid BHIS media, which consists of brain heart infusion media supplemented with yeast extract and 0.1% (w/v) *L*-cysteine [76]. BHIS media was supplemented with taurocholate (TA; 0.1% w/v), thiamphenicol (5–10 μg/mL), kanamycin (50 μg/mL), cefoxitin (16 μg/mL), FeSO_4_ (50 μM), and/or erythromycin (10 μg/mL) as indicated. Cultures were grown at 37°C, under anaerobic conditions using a gas mixture containing 85% N_2_, 5% CO_2_, and 10% H_2_.

**Table 1:** Strains and Plasmids used in this study.

Strain #	Strain name	Relevant genotype or features	Source or reference
***C*. *difficile* strains**			
11	JIR8094	*erm*-sensitive derivate of 630	[83]
35	*spo0A* ^−^	JIR8094 *spo0A*::*ermB*	[40]
47	*sleC* ^−^	JIR8094 *sleC*::*ermB*	[23]
278	*gerS* ^−^	JIR8094 *gerS*::*ermB*	This study
111	JIR8094/EV	JIR8094 carrying pMTL83151 empty vector	[23]
330	*gerS* ^−^/EV	JIR8094 *gerS*::*ermB*/pMTL83151	This study
425	*alr2* ^−^	JIR8094 *alr2*::*ermB*	This study
520	*gerS* ^−^/*gerS*	JIR8094 *gerS*::*ermB*/pMTL83151-*acpS-CD3465-gerS*	This study
574	*gerS* ^−^/single	JIR8094 *gerS*::*ermB*/pMTL83151-*gerS*	This study
630	*gerS* ^−^/ΔSP	JIR8094 *gerS*::*ermB*/pMTL83151-*acpS-CD3465*-ΔSP*gerS*	This study
632	*gerS* ^−^/C22S	JIR8094 *gerS*::*ermB*/pMTL83151-*acpS-CD3465-gerS*(C22S)	This study
***E*. *coli* strains**			
41	DH5α	F– Φ80*lacZ*ΔM15 Δ(*lacZYA-argF*) U169 *recA1 endA1 hsdR17* (rK–, mK+) *phoA supE44* λ– *thi-1 gyrA96 relA1*	D. Cameron
269	pET28a	pET28a in DH5α	M. Bogyo
373	pET22b-CPDSacI	pET22b-*CPD* encoding MARTX_Vc_ toxin 3442–3650 aa	[78]
531	HB101/pRK24	F- *mcrB mrr hsdS20*(rB- mB-) *recA13 leuB6 ara-13 proA2 lavYI galK2 xyl-6 mtl-1 rpsL20* carrying pRK24	C. Ellermeier
556	DH5α/pJS107	pJS107 in DH5α	[21]
686	pMTL83151	pMTL83151 in HB101/pK424	[23]
892	BL21(DE3)	F–*ompT hsdSB*(rB–, mB–) *gal dcm* (DE3)	Novagen
951	pJS107/*gerS*	pJS107-*gerS* targeting bp 177 in HB101/pK424	This study
982	pET22b-*cspC*_opt-CPD	pET22b-*cspC* codon optimized fused to *CPD*	This study
1112	pET28a-*gerS*	pET28a-*gerS* encoding 66–591 aa (no signal peptide)	This study
1266	pJS107-*alr2*	pJS107-*alr2* targeting bp 666 in HB101/pK424	This study
1373	pMTL83151-*gerS* (dual)	pMTL83151-*acpS-CD3465-gerS* in HB101/pK424	This study
1396	pMTL83151-single	pMTL83151-Proximal promoter-*gerS* in HB101/pK424	This study
1434	pMTL83151-C22S	pMTL83151-Dual promoter-*acpS-CD3465-gerS*(C22S) in HB101/pK424	This study
1435	pMTL83151-ΔSP	pMTL83151-Dual promoter-*acpS-CD3465-gerS*(ΔSP) in HB101/pK424	This study
**Plasmids**			
	pET28a	IPTG-inducible expression plasmid, Kan^R^	Novagen
	pMTL83151	Multi-copy complementation plasmid, Cam^R^	[84]
	pJS107	TargeTron construct based on pJIR750ai (group II intron *ermB*::RAM, *ltrA*); *catP*	[21]
	pMTL83151	pCB102, Tra^+^; *catP*	N. Minton, [84]
	pCE245	TargeTron construct based on pJIR750ai (group II intron *ermB*::RAM, *ltrA*); *catP*	C. Ellermeier

Sporulation was induced on solid media containing 70% BHIS and 30% SMC (90 g BactoPeptone, 5 g protease peptone, 1 g NH_4_SO_4_, 1.5 g Tris base, 15 g agar per liter) [77], as previously described. For strains carrying pMTL83151 derivatives, sporulation was induced on 70:30 media containing 5 μg/mL thiamphenicol.

HB101/pRK24 strains were used for conjugations and BL21(DE3) strains were used for protein production. *E*. *coli* strains (Table 1) were routinely grown at 37°C, shaking at 225 rpm in Luria-Bertani broth (LB). Media was supplemented with chloramphenicol (20 μg/mL), ampicillin (50 μg/mL), or kanamycin (30 μg/mL) as indicated.

### *E*. *coli* strain construction

*E*. *coli* strains are listed in Table 1; all primers are listed in S1 Table. For disruption of *gerS* and *alr2*, a modified plasmid containing the retargeting group II intron, pCE245 (a gift from C. Ellermeier, University of Iowa), was used as the template. Primers for amplifying the targeting sequence from the template carried flanking regions specific for each gene target and are listed as follows: *gerS* (#1122, 1123, 1124 and 532, the EBS Universal primer (Sigma Aldrich) and *alr2* (#1385, 1386, 1385 and 532). The resulting retargeting sequences were digested with BsrGI and HindIII and cloned into pJS107 [21], which is a derivative of pJIR750ai (Sigma Aldrich). The ligations were transformed into DH5α and confirmed by sequencing. The resulting plasmids were used to transform HB101/pRK24.

To construct the dual promoter complementation construct (S4 Fig), primers #1464 and 1466 were used to amplify an ~1.8 kB construct containing *acpS*, *CD3465*, *gerS*, and 360 bp upstream of *acpS* using 630 genomic DNA as the template. To construct the single promoter complementation construct, primers #1667 and 1466 were used to amplify *gerS* containing 367 bp upstream of *gerS* using 630 genomic DNA as the template. The *gerS* C22S and ΔSP complementation constructs were made using PCR splicing by overlap extension (SOE). For C22S, primer pair #1464 and 1734 was used to amplify the 5’ SOE product (containing the C22S mutation), while primer pair #1733 and 1466 was used to amplify the 3’ SOE product (containing the C22S mutation). The resulting fragments were mixed together, and flanking primers #1464 and 1466 were used to generate the dual promoter complementation construct that encodes the C22S mutation. To construct the ΔSP complementation construct, SOE primers #1464 and 1727 were used to generate a 5’ fragment; primers #1726 and 1466 were used for the 3’ SOE product. The flanking primers #1464 and 1466 were used to amplify the ΔSP complementation construct, which deletes the region encoding residues 2–22. All complementation constructs were digested with NotI and XhoI and ligated into pMTL83151 digested with the same enzymes.

To construct a strain producing GerS for antibody production, primer pairs #1278 and 1173 were used to amplify *gerS* lacking the signal peptide sequence using genomic DNA as the template. The resulting PCR products were digested with NdeI and XhoI, ligated to pET28a, and transformed into DH5α. The resulting pET28a-*gerS* plasmid was used to transform BL21(DE3) for protein production. To construct a strain for generating mouse anti-CspC antibodies, primer pairs #1128 and 1166 were used to amplify codon-optimized *cspC* using pJS148 as the template. The resulting PCR products were digested with NdeI and SacI, ligated to pET22b-CPDSacI [78], and transformed into DH5a. The resulting pET22b-*cspC*_opt-CPD was transformed into BL21(DE3) for protein production.

### Bioinformatic analyses

Homologs of *C*. *difficile* 630 GerS (CD3464) were identified using NCBI psi-blast. Homologs identified in Peptostreptococcaceae family members gave an e-value < e^-52^, whereas the next closest homolog in a *Clostridium* spp. gave an e-value > e^-27^. When GerS lacking its N-terminal signal peptide was used in the psi-blast search, the difference in e-value cut-offs was < e^-52^ for Peptostreptococcaceae family members and the next closest homolog in a *Clostridium* spp. gave an e-value > e^-23^.

### *C*. *difficile* strain construction

*C*. *difficile* strains were constructed using TargeTron-based gene disruption as described previously (S2 Fig, [40,79]). TargeTron constructs in pJS107 were conjugated into *C*. *difficile* using *E*. *coli* HB101/pRK24 as the donor strain. HB101/pRK24 strains containing the appropriate pJS107 construct were grown aerobically to exponential phase in 2.5 mL of LB supplemented with ampicillin (50 μg/mL) and chloramphenicol (10 μg/mL). Cultures were pelleted, transferred into the anaerobic chamber, and resuspended with 1.5 mL of late-exponential phase *C*. *difficile* JIR8094 cultures (grown anaerobically in BHIS broth). The resulting cell mixture was plated as seven 100 μL spots onto pre-dried, pre-reduced BHIS agar plates. After overnight incubation, all growth was harvested from the BHIS plates, resuspended in 2.5 mL pre-reduced BHIS, and twenty-one 100 μL spots per strain were plated onto three BHIS agar plates supplemented with thiamphenicol (10 μg/mL), kanamycin (50 μg/mL), and cefoxitin (16 μg/mL) to select for *C*. *difficile* containing the pJS107 plasmid. After 24–48 hrs of anaerobic growth, single colonies were patched onto BHIS agar supplemented with thiamphenicol (10 μg/mL), kanamycin (50 μg/mL), and FeSO_4_ (50 μM) to induce the ferredoxin promoter of the group II intron system. After overnight growth, patches were transferred to BHIS agar plates supplemented with erythromycin (10 μg/mL) for 24–72 hrs to select for cells with activated group II intron systems. Erythromycin-resistant patches were struck out for isolation onto the same media and individual colonies were screened by colony PCR for a 2 kb increase in the size of *gerS* (primer pair #1212 and 1173) and *alr2* (primer pair #1352 and 1359) (S2 Fig).

### *C*. *difficile* complementation

HB101/pRK24 donor strains carrying the appropriate complementation construct were grown in LB containing ampicillin (50 μg/mL) and chloramphenicol (20 μg/mL) at 37°C, 225 rpm, under aerobic conditions, for 6 hrs. *C*. *difficile* recipient strains *gerS* ^−^ and *alr2* ^−^ containing group II intron disruptions, were grown anaerobically in BHIS broth at 37°C with gentle shaking for 6 hrs. HB101/pRK24 cultures were pelleted at 2500 rpm for 5 min and the supernatant was removed. Pellets were transferred to the anaerobic chamber and gently resuspended in 1.5 mL of the appropriate *C*. *difficile* culture. The resulting mixture was inoculated onto pre-dried, pre-reduced BHIS agar plates, as seven 100 μL spots for 12 hrs. All spots were collected anaerobically and resuspended in 1 mL PBS. One hundred microliters of the resulting suspension was spread onto pre-dried, pre-reduced BHIS agar plates supplemented with thiamphenicol (10 μg/mL), kanamycin (50 μg/mL), and cefoxitin (10 μg/mL), five plates per conjugation. Plates were monitored for colony growth for 24–72 hrs. Individual colonies were struck out for isolation and analyzed for complementation using the heat resistance assay to test for functional spore formation and Western blot analysis. A minimum of two independent clones from each complementation strain was phenotypically characterized.

### Sporulation

*C*. *difficile* strains were grown from glycerol stocks on BHIS plates supplemented with TA (0.1% w/v), or with TA and thiamphenicol (5 μg/mL) for strains carrying pMTL83151-derived vectors. Colonies that arose on BHIS agar plates were then used to inoculate 70:30 agar plates containing 5 μg/mL thiamphenicol for 17–24 hrs depending on the assay. Sporulating cells were harvested into PBS, pelleted, and resuspended in PBS for visualization by phase contrast microscopy and further processing as needed.

### Heat resistance assay

*C*. *difficile* strains were induced to sporulate as described above and functional (heat-resistant) spore formation was measured as previously described [41] with the following exceptions. After 24 hrs of growth, cells were harvested into 600 μL of pre-reduced PBS. The sample was divided into two tubes. One tube was exposed to 60°C for 25–30 minutes. Heat-treated and untreated cells were serially diluted, and dilutions were plated on pre-reduced BHIS-TA plates. After ~20 hrs colonies were counted, and cell counts were determined. The percent of heat-resistant spores was determined based on the ratio of heat-resistant cells to total cells, and heat-resistance efficiencies were determined based on the ratio of heat-resistant cells for a strain compared to wildtype. Results are based on a minimum of three biological replicates. The raw data for the heat resistance assay is provided in S2 Table.

### Spore purification

Sporulation was induced by growing *C*. *difficile* strains on 70:30 plates (with 5 μg/mL thiamphenicol when appropriate for 2–3 days, and spores were harvested in ice-cold water as previously described [23,76] with the following modifications. Spores were incubated on ice overnight following multiple water washes. The following day, they were pelleted and treated with DNase (New England Biolabs) at 37°C for 30 minutes. Following DNAse treatment, the spores were purified on a HistoDenz (Sigma Aldrich) gradient, evaluated for purity by phase contrast microscopy, and the optical density of the suspension was measured at OD_600_. Spores were stored in water at 4°C.

### Germination assay

Approximately 1 x 10^7^ spores (equivalent of 0.35 OD_600_ units) were re-suspended in 100 μL of water. Ten microliters of the suspension was serially diluted in PBS, and dilutions were plated onto pre-reduced BHIS-TA. After ~22 hrs, colonies arising from germinated spores were counted. Germination efficiency represents the number of CFUs produced by germinating spores of a given strain relative to wild type. Results are based on a minimum of three biological replicates. The remaining 90 μL of the spores were pelleted and resuspended in EBB buffer for Western blot analyses.

To assess the effect of heat treatment on spore viability, the procedure above was followed, with the exception that 2 x 10^7^ spores were re-suspended in 200 μL of water (equivalent of 0.7 OD_600_ units) and the sample was divided into two. One half was incubated at 60°C for 30 min, while the other half was left untreated.

The effect of taurocholate concentration on spore germination efficiency was determined by re-suspending ~4 x 10^7^ spores (~1.4 OD_600_ units) in 160 μL of water in triplicate. Two hundred microliters of BHIS was added to each spore suspension. Ninety microliters of this suspension was added to either 10 μL of water, 0.1% TA, 1% TA, or 10% TA (to give a final concentration of 0.01% TA, 0.1% TA, or 1% TA). The samples were incubated for 20 min at 37°C, and a 10 μL aliquot was removed for 10-fold serial dilutions into PBS. Ten microliters of the serial dilutions were plated on BHIS to determine the number of spores that had initiated germination. The serial dilutions for untreated and 1% TA-treated spores were also plated on BHIS-TA plates to determine the maximum level of spore germination. Spore germination was maximal following exposure to 1% TA. The remaining samples were pelleted for Western blot analysis.

### Antibody production

The anti-GerS used in this study was raised against GerS-His_6_ lacking its signal peptide in rabbits by Cocalico Biologicals (Reamstown, PA). The anti-CspC mouse antibodies were raised against recombinant untagged CspC in mice by Cocalico Biologicals (Reamstown, PA). GerS-His_6_ was purified from *E*. *coli* strains #1112 using Ni^2+^-affinity resin as previously described [23]. Recombinant, untagged CspC was purified using the autoprocessing CPD tag as previously described [78] followed by gel filtration [23].

### Western blot analyses

*C*. *difficile* cell pellets were processed as previously described [40,60]. Briefly, pellets were freeze-thawed three times, diluted in EBB buffer (8 M urea, 2 M thiourea, 4% (w/v) SDS, 2% (v/v) β-mercaptoethanol), and incubated at 95°C for 20 min with vortexing every 5 min. *C*. *difficile* spores (~1 x 10^6^) were resuspended in EBB buffer, which can extract proteins in all layers of the spore including the core. Samples were centrifuged for 5 min at 15,000 rpm and 4X sample buffer (40% (v/v) glycerol, 1 M Tris pH 6.8, 20% (v/v) β-mercaptoethanol, 8% (w/v) SDS, and 0.04% (w/v) bromophenol blue), was added. Samples were incubated again at 95°C for 5–15 minutes with vortexing followed by centrifugation for 5 min at 15,000 rpm. The samples were resolved by SDS-PAGE and transferred to Millipore Immobilon-FL membrane. The membranes were blocked in Odyssey Blocking Buffer. Rabbit polyclonal rabbit anti-GerS or anti-GPR [40] antibodies were used at a 1:1,000 dilution; anti-CspB [23] antibodies were used at a 1:2,500 dilution, and the anti-SleC [23] antibody was used at a 1:5,000 dilution. Polyclonal mouse anti-SpoIVA [80] and anti-CspC antibodies were used at 1:2,500 dilutions. IRDye 680CW and 800CW infrared dye-conjugated secondary antibodies were used at 1:20,000 dilutions. The Odyssey LiCor CLx was used to detect secondary antibody infrared fluorescence emissions.

### RNA processing

RNA from WT/EV, *gerS* ^−^/EV, *gerS* ^−^/*gerS*, *gerS* ^−^/C22S, and *gerS* ^−^/ΔSP strains grown for 24 hrs on 70:30 sporulation media containing thiamphenicol (5 μg/mL) was extracted for qRT-PCR analyses of the *gerS* transcript. RNA was extracted using a FastRNA Pro Blue Kit (MP Biomedical) and a FastPrep-24 automated homogenizer (MP Biomedical). Contaminating genomic DNA was depleted using three successive DNase treatments, and mRNA enrichment was done using an Ambion MICROB*Express* Bacterial mRNA Enrichment Kit (Invitrogen). Samples were tested for genomic DNA contamination using quantitative PCR for *rpoB*. Enriched RNA was reverse transcribed using Super Script First Strand cDNA Synthesis Kit (Invitrogen) with random hexamer primers.

### Quantitative RT-PCR

Transcript levels of *gerS* and *rpoB* (housekeeping gene) were determined from cDNA templates prepared from 3 biological replicates of WT/EV, *spo0A* ^−^/EV, *gerS* ^−^/EV, *gerS* ^−^/*gerS*, *gerS* ^−^/C22S, and *gerS* ^−^/ΔSP strains. Gene-specific primer pairs for *gerS* (#1278 and #1173), *alr2* (#1668 and #1356), and *rpoB* [40] were used. Quantitative real-time PCR was performed (as described by [41]. Briefly, using Maxima^TM^ SYBR^TM^ Green qPCR Master Mix (Thermo Scientific), 50 nM of gene specific primers, and an ABI PRISM 7900HT Sequence Detection System (Applied Biosystems). Transcript levels were normalized to the housekeeping gene *rpoB* using the standard curve method.

### Fluorescence microscopy

For live cell fluorescence microscopy studies, *C*. *difficile* strains were harvested in PBS, pelleted, and resuspended in PBS. For characterization of mutant phenotypes, cells were resuspended in PBS containing 1 μg/mL FM4-64 (Molecular Probes) and 15 μg/mL Hoechst 33342 (Molecular Probes). All live bacterial suspensions (4 μL) were added to a freshly prepared 1% agarose pad on a microscope slide, covered with a 22 x 22 mm #1 coverslip and sealed with VALAB (1:1:1 of vaseline, lanolin, and beeswax) as previously described [41,81].

DIC and fluorescence microscopy was performed using a Nikon PlanApo Vc 100x oil immersion objective (1.4 NA) or a Nikon PlanApo Vc 60x oil immersion objective (1.4 NA) on a Nikon Eclipse Ti2000 epifluorescence microscope. Multiple fields for each sample were acquired with an EXi Blue Mono camera (QImaging) with a hardware gain setting of 1.0 and driven by NIS-Elements software (Nikon). Images were subsequently imported into Adobe Photoshop CS6 for minimal adjustments in brightness/contrast levels and pseudocoloring.

### Artificial germination assay

Artificial germination was determined using thioglycollate and lysozyme treatment. About 1 x 10^6^ spores were pelleted at 8,000 RPM for 3 min, resuspended in 250mM thioglycollate, and incubated at 50°C for 30 min based on previously methods developed [21,58]. Spores were washed with 150 μL of PBS, pelleted, resuspended in 150 μL (2 mg/mL) lysozyme and incubated at 37°C for 15 min. Equivalent numbers of spores for each strain were incubated at the indicated temperatures without thioglycollate or lysozyme treatment for the untreated sample. The spore samples were plated on either BHIS or BHIS-TA. Natural germination represents the number of spores in the untreated sample that outgrew to form colonies on BHIS-TA media. Artificial Germination represents the number of thioglycollate/lysozyme-treated spores that germinated and outgrew to form colonies on BHIS media.

### Decoating assay

About 1 x 10^7^ spores (~0.35 OD_600_) were pelleted and resuspended in 30 μL of decoat buffer (0.1 M H_3_BO_3_ pH 10.0, 1% SDS, 2% β-ME) [57]. The sample was incubated for 30 min at 37°C and then pelleted. The supernatant, representing the “coat-extractable” (CE) fraction, was removed, and the pellet was washed in 20 μL decoat buffer and incubated for 10 min. The sample was re-pelleted, and the supernatant was added to the CE; 40 μL of EBB was added to the pooled fractions. The decoated spores were re-suspended in 90 μL EBB to produce the cell lysate (pellet) fraction. For the input fraction, representing whole spore lysate, equal numbers of spores were pelleted and resuspended in 90 μL EBB. All samples were boiled for a minimum of 15 min, followed by centrifugation and sample resuspension. Fractions were pelleted one more time before the samples were resolved by SDS-PAGE and analyzed by Western blotting as described above.

### Cortex hydrolysis assay

Cortex hydrolysis was analyzed by transmission electron microscopy for untreated spores (0’) and 15’ and 45’ after germinant addition. About 4 x 10^7^ spores (1.4 OD_600_ units) were resuspended in 160 μL of water in triplicate. Two hundred microliters of BHIS was added to each spore suspension. Forty microliters of water was added to one sample, while 40 μL of 10% taurocholate (w/v) was added to the remaining samples. The spores were incubated under anaerobic conditions for the indicated time point after which a small sample was removed for visualizing by phase-contrast microscopy and plating on BHIS and BHIS-TA. The remainder of the sample was pelleted and re-suspended in osmium tetroxide fixative for TEM analysis as previously described [60]. TEM grids for each sample analyzed were prepared as previously described [41]. A minimum of 50 spore pictures chosen at random were analyzed for each strain observed. To account for asymmetrical spore shapes, two orthologous cortex lengths were measured such that a minimum and maximum cortex thickness was obtained for every spore. Cortex length was defined as the distance between the outer most germ cell wall and the cortex outer edge. The minimum and maximum measurements were averaged for each spore and the upper and lower values were discarded. The cortex length reported represents the average of these measurements.

### Ca-DPA release assay

To evaluate the amount of Ca-DPA released in response to germinant relative to the total amount of Ca-DPA observed in the spore core, a modified Ca-DPA release assay was adopted from [21]. About 2x10^7^ spores from each strain were re-suspended in (i) 1 mL of germination buffer (0.3 m M (NH_4_)_2_SO_4_, 6.6 mM KH_2_PO_4_, 15 mM NaCl, 59.5 mM NaHCO_3_, and 35.2 mM Na_2_HPO_4_) and incubated at 37°C for 30 min (background); (ii) 1 mL of germination buffer containing 10% freshly prepared taurocholate and 10 mM glycine and incubated at 37°C for 30 min (DPA release); (iii) 1 mL of germination buffer and incubated at 100°C for 1 hr (total DPA). After incubation, samples were spun down at 15,000 RPM for 2 min. 700 μL was transferred to UV clear cuvettes, and the A_270_ was determined. The % Ca-DPA release was determined by subtracting the background DPA release value from the germinant containing DPA release value and dividing by total DPA. Total DPA measured in wild type was set as 100% total DPA.

### O.D. kinetics assay

Approximately 1 x 10^7^ spores (0.48 OD_600_ units) were resuspended in BHIS to a total volume of 1100. The sample was divided into two: 540 μL was added to a cuvette containing 60 μL of water, while the other sample was added to a cuvette containing 60 μL of 10% taurocholate. The samples were mixed, and the OD_600_ was measured every 3–6 mins for 45 min.

### Virulence studies

All animal studies were performed with prior approval from the Texas A&M University Institutional Animal Care and Use Committee. Female Syrian golden hamsters (80–120 g) were housed and tested for *C*. *difficile* susceptibility as previously described [21,82]. To induce *C*. *difficile* infection, hamsters were gavaged with 30 mg/kg clindamycin prior to *C*. *difficile* spore inoculum. After 5 days, 10 hamsters per *C*. *difficile* strain tested were gavaged with 1,000 spores and monitored for signs of disease. Hamsters showing disease symptoms (wet tail, poor fur coat and lethargy) were euthanized by CO_2_ asphyxia followed by thoracotomy as a secondary means of death.

### Ethics statement

All animal procedures were performed with prior approval from the Texas A&M Institutional Animal Care and Use Committee under the approved Animal Use Protocol number 2014–0085. Animals showing signs of disease were euthanized by CO_2_ asphyxia followed by thoracotomy as a secondary means of death, in accordance with Panel on Euthanasia of the American Veterinary Medical Association. Texas A&M University’s approval of Animal Use Protocols is based upon the United States Government’s Principles for the Utilization and Care of Vertebrate Animals Used in Testing, Research and Training and complies with all applicable portions of the Animal Welfare Act, the Public Health Service Policy for the Humane Care and Use of Laboratory Animals, and all other federal, state, and local laws which impact the care and use of animals.

## Supporting Information

S1 Fig Representative images of Integrated Genome Viewer software [87] used to visualize RNA-Seq data [41]. Histograms of RNA sequence reads obtained for the indicated strains are shown in grey. The direction of transcription is indicated by the angle bracket. *gerS* and *alr2* are under the control of mother cell-specific σ^E^ [40,41,61].
(TIF)

S2 Fig Generation of *gerS* ^−^ and *alr2* ^−^ strains using Targetron gene disruption. (A) Schematic of the Group II Intron system [79] used for insertional mutagenesis of *gerS* and *alr2*. (B) Colony PCR of wildtype, *gerS* ^−^, and *alr2* ^−^ strains using primers that flank the gene of interest. The Group II Intron is ~2 kb.
(TIF)

S3 Fig Sporulation of *gerS* ^−^ and *alr2* ^−^ strains is similar to wild type. Fluorescence microscopy analysis of wild type, *spo0A* ^−^, *gerS* ^−^, and *alr2* ^−^ sporulating cells. Strains were grown on sporulation media for 20 hrs and visualized by live differential interference contrast (DIC). The nucleoid was stained with Hoechst (blue), and membranes were stained with FM4-64 (red). Orange arrows designate forespores that have not progressed beyond asymmetric division (flat polar septa); blue arrows designate cells that stain with both Hoechst and FM4-64; yellow arrows designate forespore compartments that exclude Hoechst but stain with FM4-64; and white arrows designate forespores that are DIC-bright and exclude both Hoechst and FM4-64. Scale bars represent 5 μm.
(TIF)

S4 Fig Plasmid complementation of *gerS* ^−^. (A) Schematic of constructs used to assay for *gerS* ^−^ complementation. Dual (*gerS*) designates a complementation construct that includes 2 potential *gerS* promoters and the upstream genes *acpS* and *CD3465*. The P_1_ promoter has been mapped by RNA-Seq transcriptional start site mapping [42]. Single designates a complementation construct where *gerS* transcription is driven from the P_1_ promoter alone. (B) Western blot analyses of *gerS* ^−^ complementation strains grown on sporulation media for 22 hrs. The strains carry empty vector (EV) or the indicated complementation constructs. The efficiency of heat-resistant spore formation was determined for each strain relative to wildtype from three biological replicates. H.R. = heat resistance.
(TIF)

S5 Fig *gerS* ^−^ does not release Ca-DPA in response to germinant. Spores isolated from wildtype carrying empty vector (WT/EV) and *gerS* ^−^ carrying empty vector (*gerS* ^−^/EV) or a *gerS* complementation construct (*gerS* ^−^/*gerS*) were analyzed for total Ca-DPA content and Ca-DPA release in response to taurocholate germinant and glycine co-germinant. The amount of Ca-DPA released by wildtype spores after boiling for 1 hr was set to 100% total Ca-DPA. Percent Ca-DPA release represents the A_270_ value after response to germinant incubation relative to the total DPA value obtained for a given strain. The results represent the average of 4 biological replicates.
(TIF)

S6 Fig *gerS* transcript levels in *gerS* ^−^ complementation strains. Transcript levels of the *gerS* were analyzed by qRT-PCR for RNA isolated from wildtype carrying empty vector (WT/EV) or *gerS* ^−^ carrying either empty vector (*gerS* ^−^/EV) or the indicated complementation constructs induced to sporulate for 24 hrs. Transcript levels were normalized to the housekeeping gene *rpoB* using the standard curve method. Data represents the average of three biological replicates. Error bars indicate the standard error of the mean. n.a. indicates not applicable, since the region amplified spans the disrupted *gerS* gene.
(TIF)

S7 Fig Effect of heat-treatment on *gerS* ^−^ spore germination. Spores isolated from wildtype carrying empty vector (WT/EV) or *gerS* ^−^ carrying either empty vector or the indicated complementation constructs were heat-treated for 30 min at 60°C prior to plating on germination media. No statistically significant changes occurred between untreated (–) or heat-treated (+) spores for a given strain with the exception of *gerS* ^−^ spores carrying empty vector (*gerS* ^−^/EV). Results represent the average of three biological replicates (** p < 0.01).
(TIF)

S8 Fig Comparison of germination initiation rates in *gerS* complementation strains. Purified spores from the indicated strains were re-suspended in BHIS. Germination was induced by the addition of taurocholate (1% final concentration). The ratio of the OD_600_ at a given time relative to the OD_600_ at time zero is plotted. The data represent the average of three independent experiments, and error bars indicate the standard deviation for each time point measured.
(TIF)

S1 Table Primers used in this study. (DOCX)

S2 Table Raw data from heat-resistant spore formation assays. (DOCX)
