# Supplementary figures and images for: Identification of a Novel Lipoprotein Regulator of Clostridium difficile Spore Germination
Source: PLoS Pathog. 2015 Oct 23;11(10):e1005239. doi: 10.1371/journal.ppat.1005239 (PMC4619724; doi:10.1371/journal.ppat.1005239)

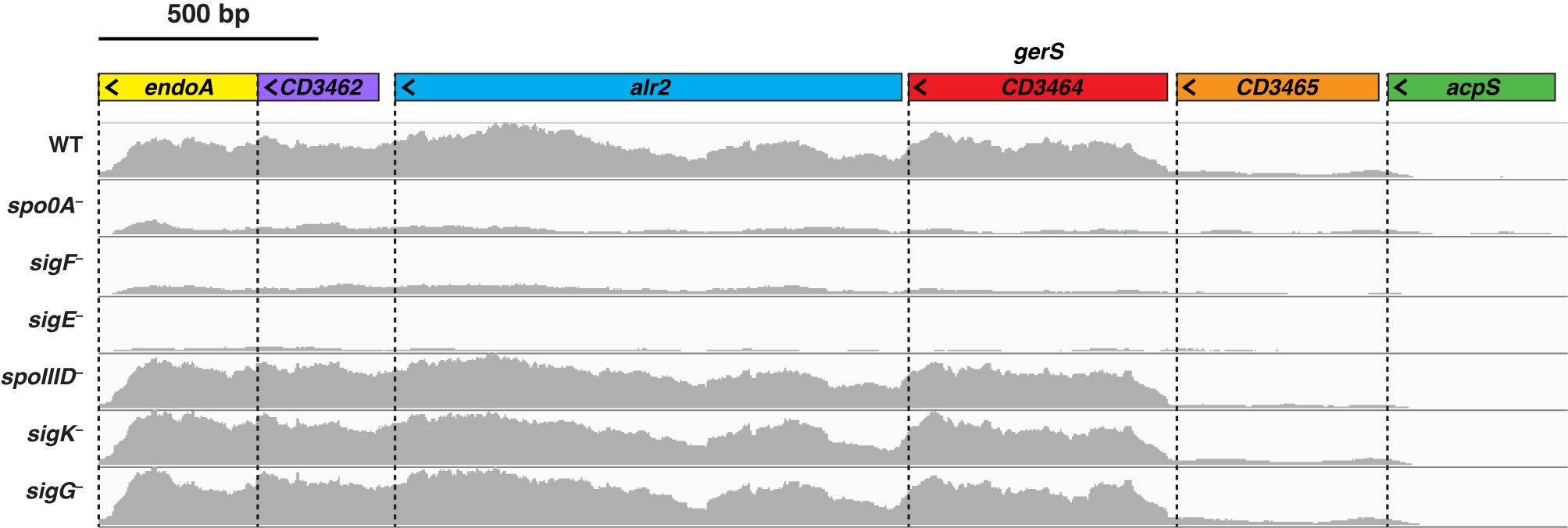

Supplement: S1 Fig — Histograms of RNA sequence reads obtained for the indicated strains are shown in grey. The direction of transcription is indicated by the angle bracket. gerS and alr2 are under the control of mother cell-specific σE [40,41,61]. (TIF) [file ppat.1005239.s001.tif]

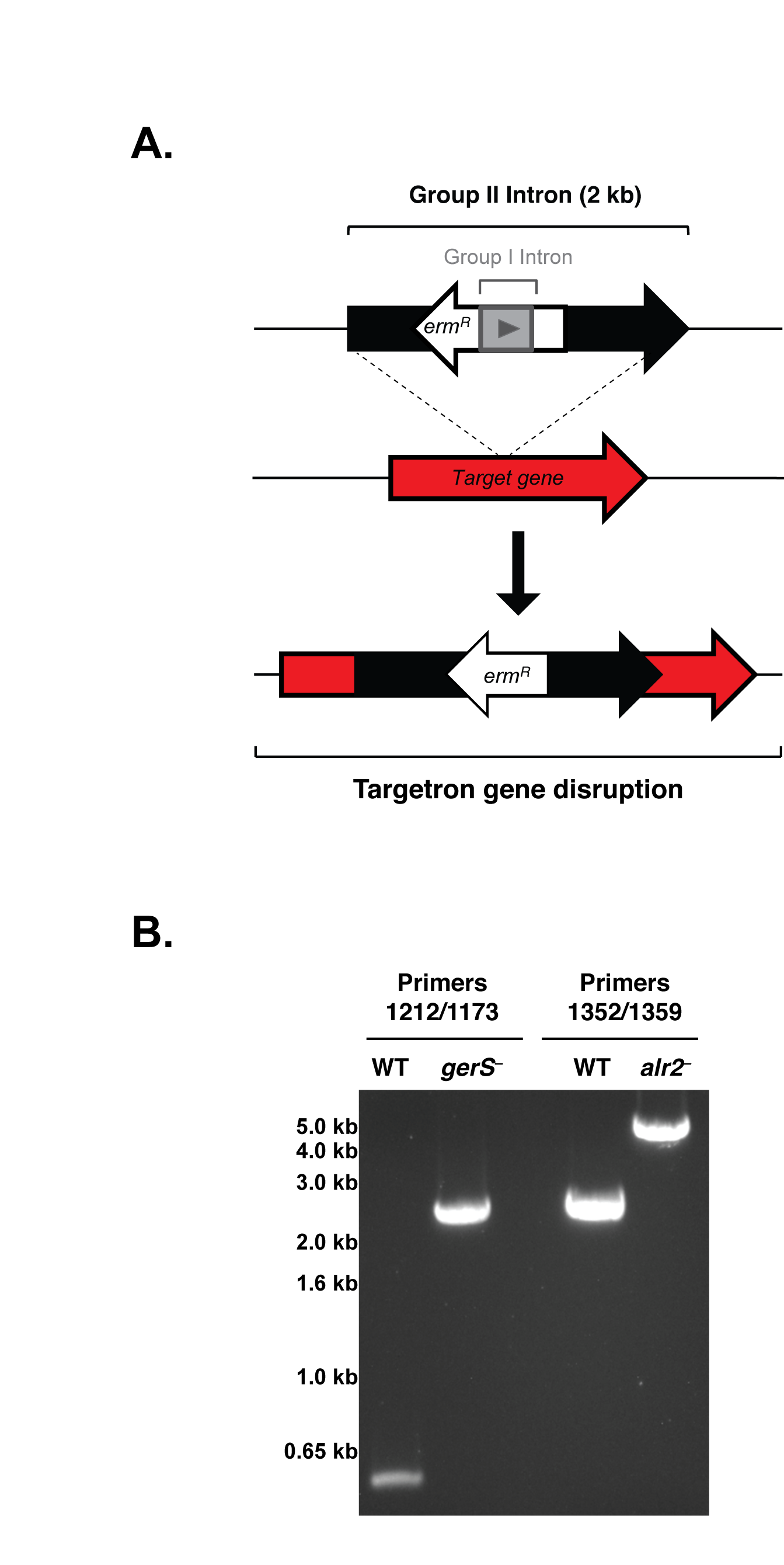

Supplement: S2 Fig — (A) Schematic of the Group II Intron system [79] used for insertional mutagenesis of gerS and alr2. (B) Colony PCR of wildtype, gerS −, and alr2 − strains using primers that flank the gene of interest. The Group II Intron is ~2 kb. (TIF) [file ppat.1005239.s002.tif]

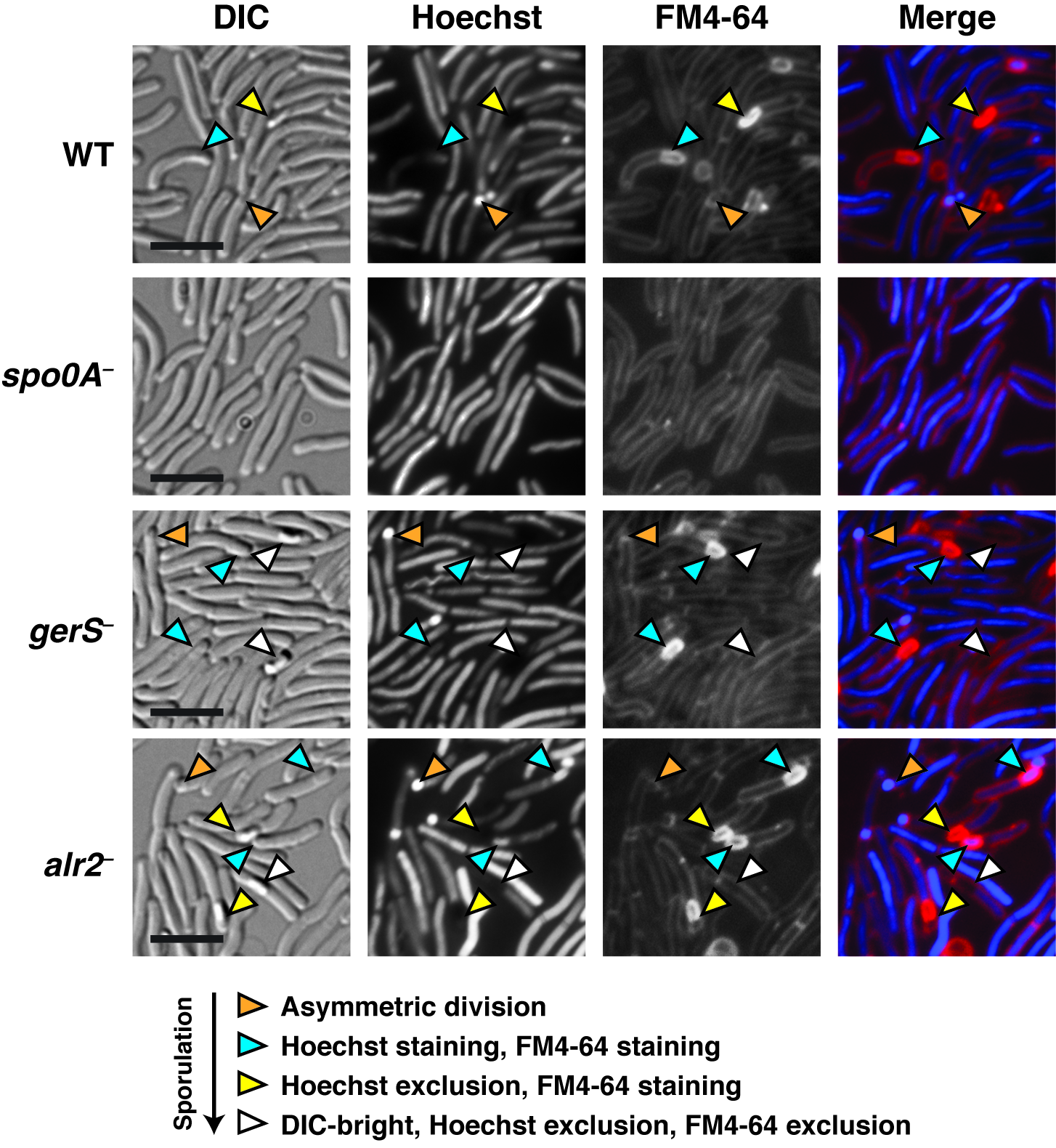

Supplement: S3 Fig — Fluorescence microscopy analysis of wild type, spo0A −, gerS −, and alr2 − sporulating cells. Strains were grown on sporulation media for 20 hrs and visualized by live differential interference contrast (DIC). The nucleoid was stained with Hoechst (blue), and membranes were stained with FM4-64 (red). Orange arrows designate forespores that have not progressed beyond asymmetric division (flat polar septa); blue arrows designate cells that stain with both Hoechst and FM4-64; yellow arrows designate forespore compartments that exclude Hoechst but stain with FM4-64; and white arrows designate forespores that are DIC-bright and exclude both Hoechst and FM4-64. Scale bars represent 5 μm. (TIF) [file ppat.1005239.s003.tif]

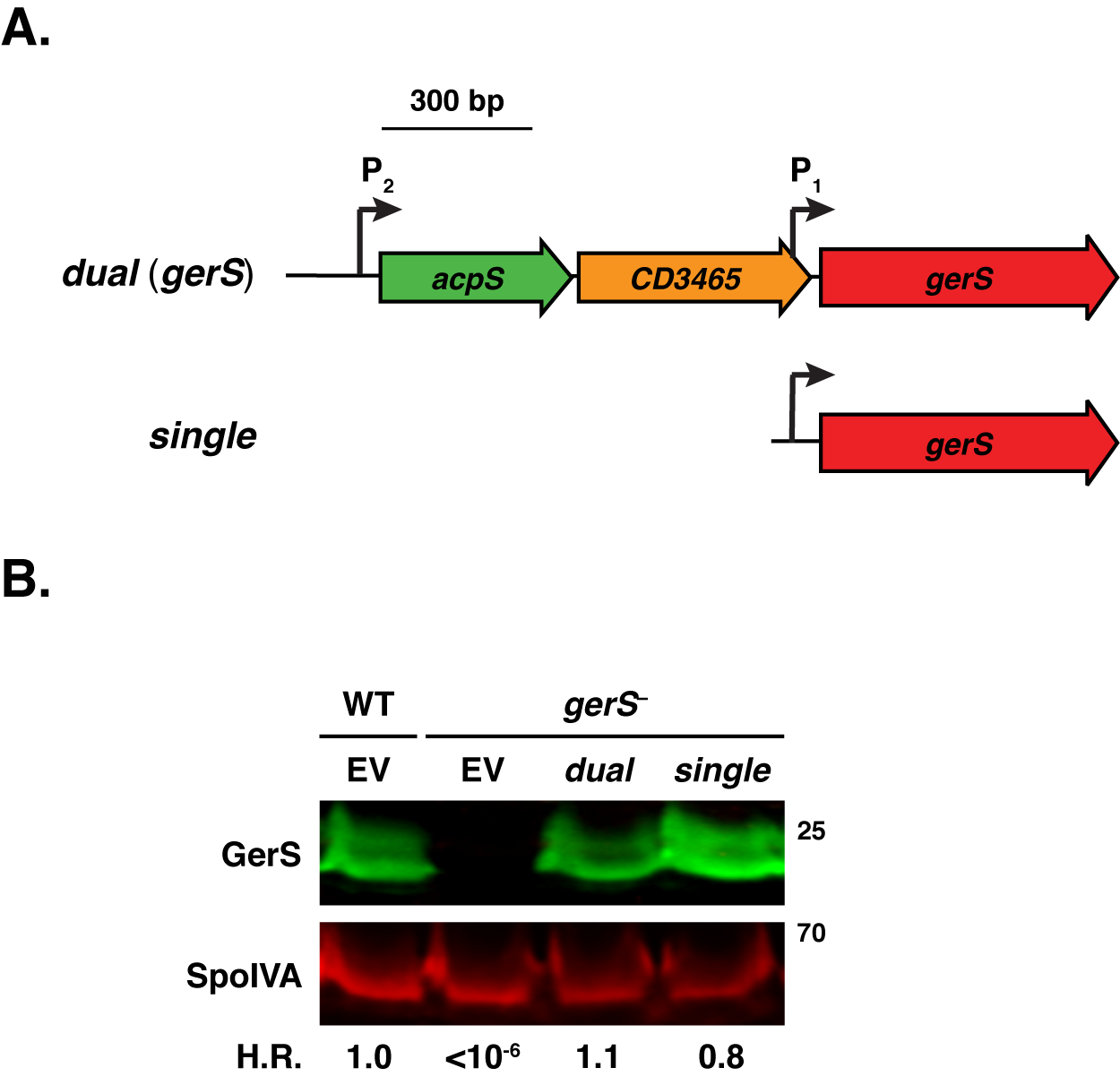

Supplement: S4 Fig — (A) Schematic of constructs used to assay for gerS − complementation. Dual (gerS) designates a complementation construct that includes 2 potential gerS promoters and the upstream genes acpS and CD3465. The P1 promoter has been mapped by RNA-Seq transcriptional start site mapping [42]. Single designates a complementation construct where gerS transcription is driven from the P1 promoter alone. (B) Western blot analyses of gerS − complementation strains grown on sporulation media for 22 hrs. The strains carry empty vector (EV) or the indicated complementation constructs. The efficiency of heat-resistant spore formation was determined for each strain relative to wildtype from three biological replicates. H.R. = heat resistance. (TIF) [file ppat.1005239.s004.tif]

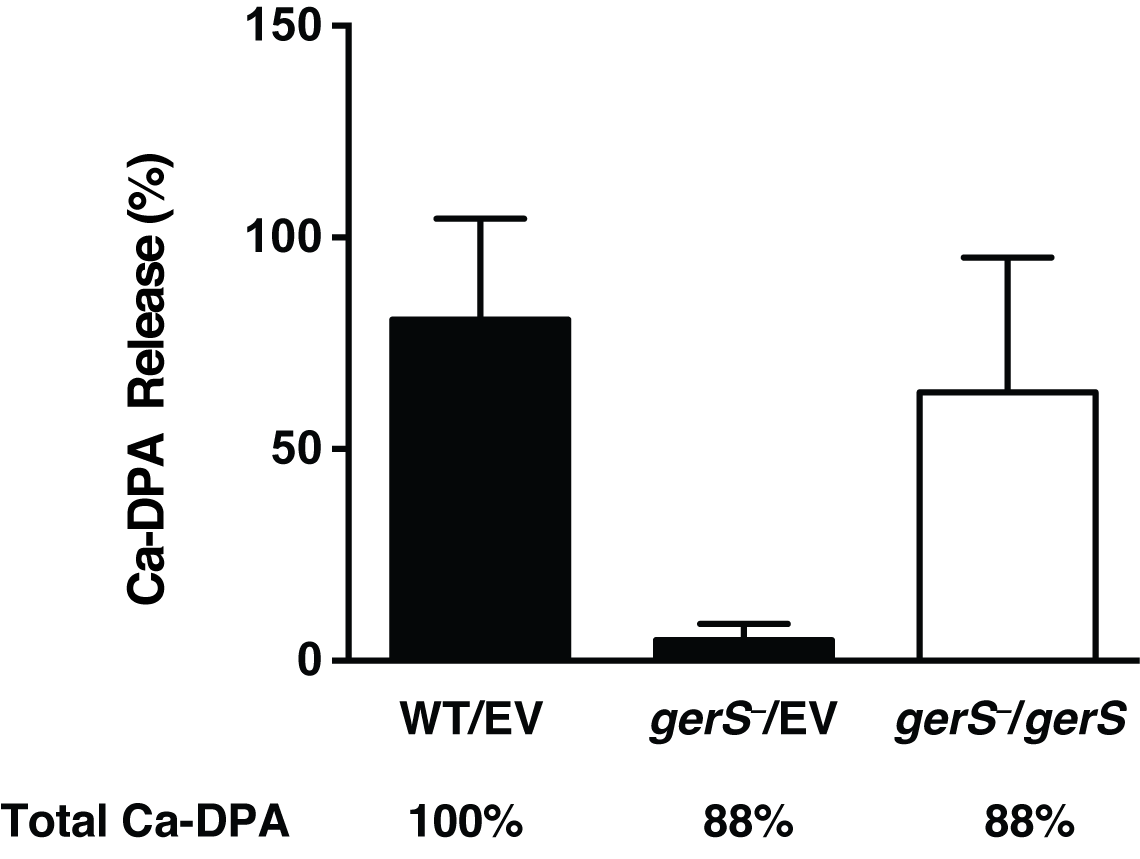

Supplement: S5 Fig — Spores isolated from wildtype carrying empty vector (WT/EV) and gerS − carrying empty vector (gerS −/EV) or a gerS complementation construct (gerS −/gerS) were analyzed for total Ca-DPA content and Ca-DPA release in response to taurocholate germinant and glycine co-germinant. The amount of Ca-DPA released by wildtype spores after boiling for 1 hr was set to 100% total Ca-DPA. Percent Ca-DPA release represents the A270 value after response to germinant incubation relative to the total DPA value obtained for a given strain. The results represent the average of 4 biological replicates. (TIF) [file ppat.1005239.s005.tif]

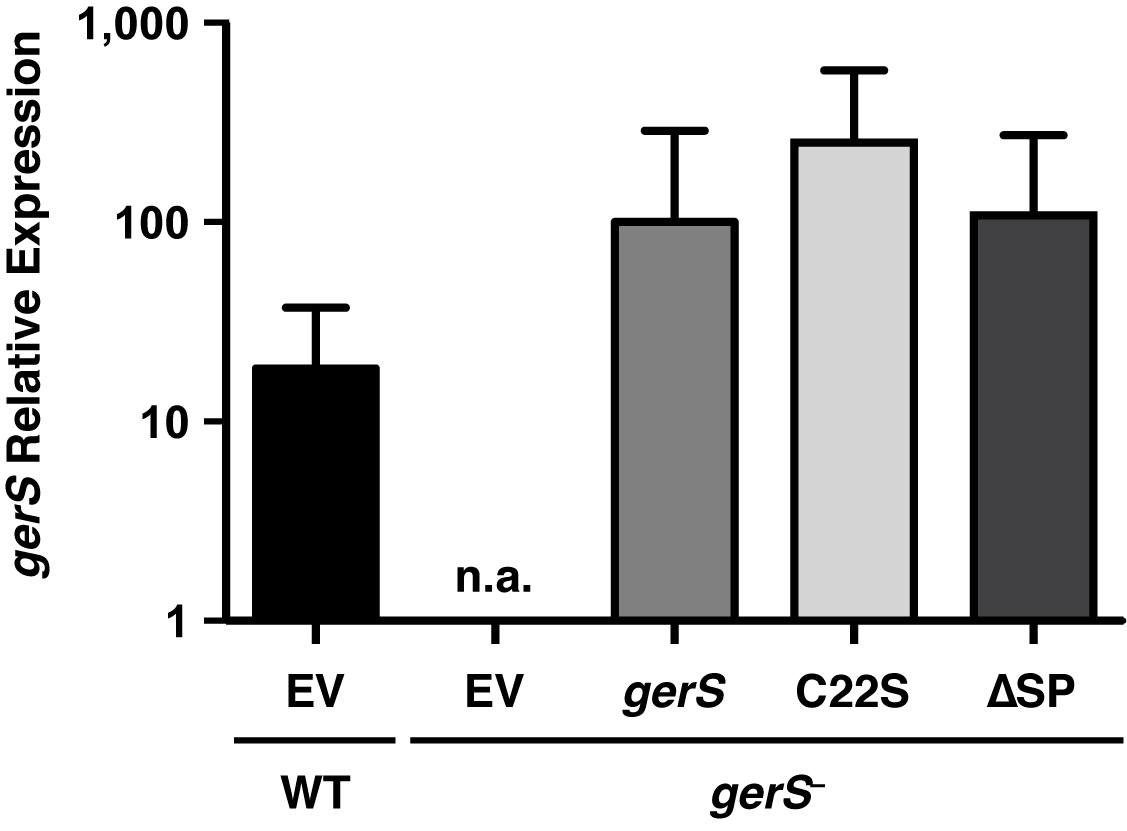

Supplement: S6 Fig — Transcript levels of the gerS were analyzed by qRT-PCR for RNA isolated from wildtype carrying empty vector (WT/EV) or gerS − carrying either empty vector (gerS −/EV) or the indicated complementation constructs induced to sporulate for 24 hrs. Transcript levels were normalized to the housekeeping gene rpoB using the standard curve method. Data represents the average of three biological replicates. Error bars indicate the standard error of the mean. n.a. indicates not applicable, since the region amplified spans the disrupted gerS gene. (TIF) [file ppat.1005239.s006.tif]

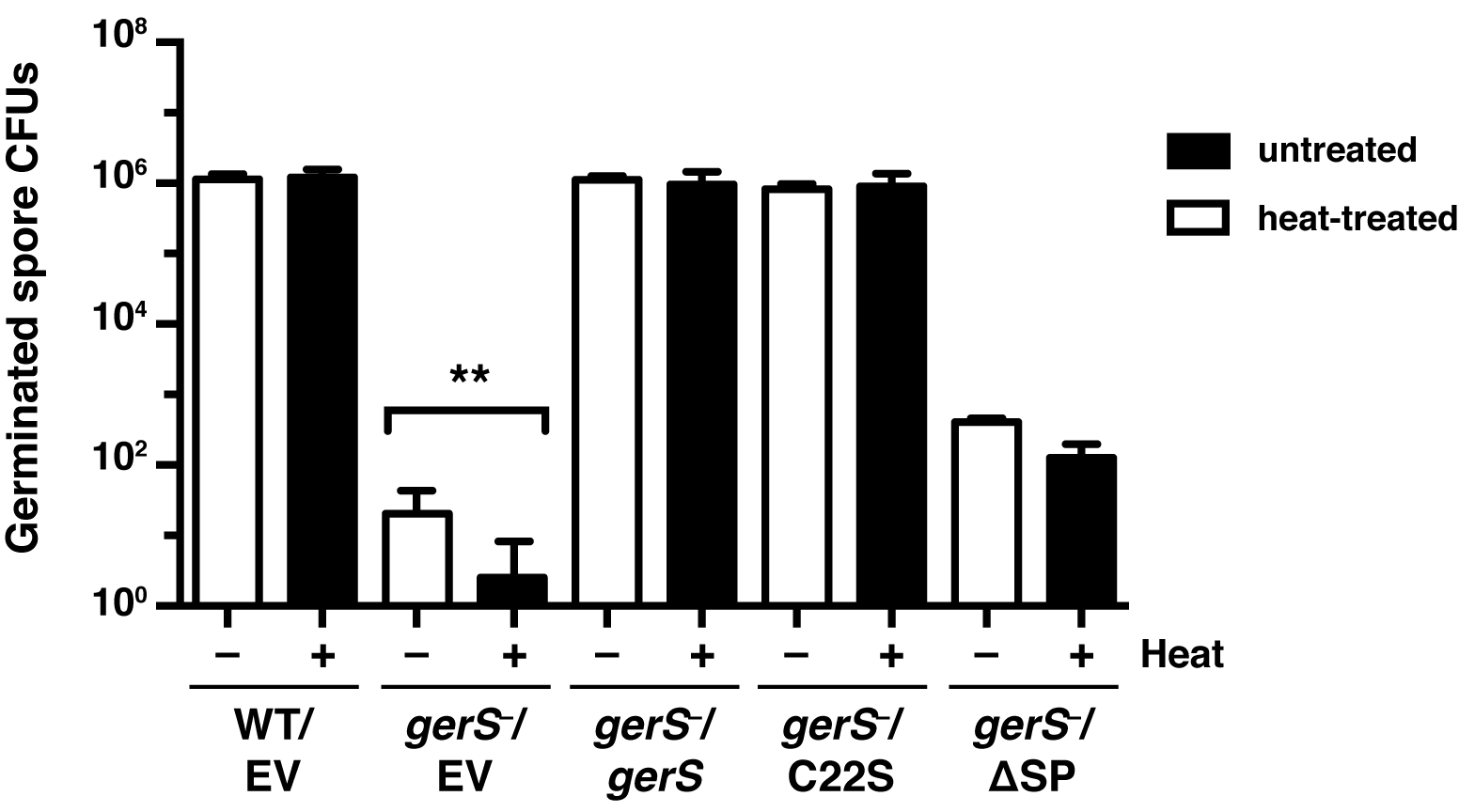

Supplement: S7 Fig — Spores isolated from wildtype carrying empty vector (WT/EV) or gerS − carrying either empty vector or the indicated complementation constructs were heat-treated for 30 min at 60°C prior to plating on germination media. No statistically significant changes occurred between untreated (–) or heat-treated (+) spores for a given strain with the exception of gerS − spores carrying empty vector (gerS −/EV). Results represent the average of three biological replicates (** p < 0.01). (TIF) [file ppat.1005239.s007.tif]

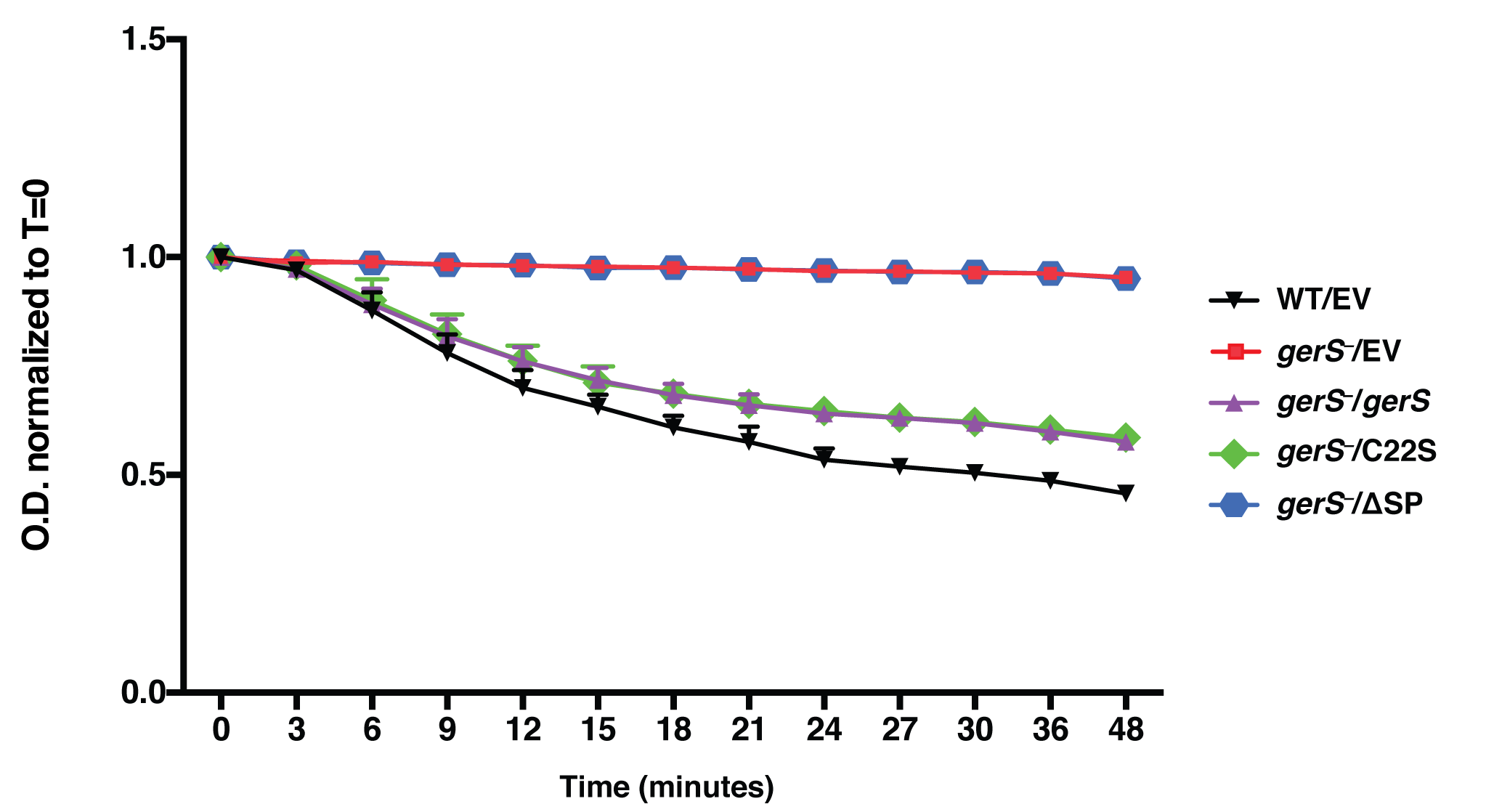

Supplement: S8 Fig — Purified spores from the indicated strains were re-suspended in BHIS. Germination was induced by the addition of taurocholate (1% final concentration). The ratio of the OD600 at a given time relative to the OD600 at time zero is plotted. The data represent the average of three independent experiments, and error bars indicate the standard deviation for each time point measured. (TIF) [file ppat.1005239.s008.tif]
