# Supplementary material for: Identification of a Novel Lipoprotein Regulator of Clostridium difficile Spore Germination
Source: PLoS Pathog. 2015 Oct 23;11(10):e1005239. doi: 10.1371/journal.ppat.1005239 (PMC4619724; doi:10.1371/journal.ppat.1005239)
Supplement: S1 Table — (DOCX) [file ppat.1005239.s009.docx]

**Table S3. Primers used in this study.**

| **Primer** | **Name** | **Sequence** |
| --- | --- | --- |
| 532 | 3’ Universal EBS | CGAAATTAGAAACTTGCGTTCAGTAAAC |
| 1122 | 5’ IBSI *gerS* 177 | AAAAAAGCTTATAATTATCCTTAATAATCGTGTGGGTGCGCCCAGATAGGGTG |
| 1123 | 3’ EBS1d *gerS* 177 | CAGATTGTACAAATGTGGTGATAACAGATAAGTCGTGTGGGCTAACTTACCTTTCTTTGT |
| 1124 | 5’ EBS2 *gerS* 177 | TGAACGCAAGTTTCTAATTTCGGTTATTATCCGATAGAGGAAAGTGTCT |
| 1128 | 5' NdeI cspC codon opt pJS148 | AAAAAA**CATATG**GAGAAAAGCTACTGCATTATTTATC |
| 1166 | 3' SacI cspC codon opt no TAA | AAAT**GAGCTC**AAGGGTGTTGGCAATCTGCTG |
| 1173 | 3’ XhoI *gerS* no stop | AATA**CTCGAG**GTTTCTGTATTCAAAATC |
| 1212 | 5’ NdeI *gerS* | AATA**CATATG**AGAAAAAAGTGGACCATAG |
| 1278 | 5’ NdeI *gerS* truncation | AGC**CATATG**CAAAAACGACAGTCCAC |
| 1352 | 5’ *CD3465*-*gerS* (981 operon) | GGATTGATTTTCTTAGATGAC |
| 1359 | 3’ *CD3462*-*CD3461* (3394 operon) | CAGAACAGGTCTAACGCC |
| 1385 | 5’ IBS1 *alr2* 666 | AAAAAAGCTTATAATTATCCTTAGATTTCAGATTAGTGCGCCCAGATAGGGTG |
| 1386 | 3’ EBS1d *alr2* 666 | CAGATTGTACAAATGTGGTGATAACAGATAAGTCAGATTAAATAACTTACCTTTCTTTGT |
| 1387 | 5’ EBS2 *alr2* 666 | TGAACGCAAGTTTCTAATTTCGATTAAATCTCGATAGAGGAAAGTGTCT |
| 1464 | 5’ NotI *gerS* operon promoter | AAAA**GCGGCCGC**AGTTTTTAGTGATAATAAATTC |
| 1466 | 3’ XhoI *gerS* | AAAA**CTCGAG**GTCTTTAGTCCCCCTTAGTTTCTG |
| 1667 | 5’ NotI *gerS*-*CD3463* operon | AAA**GCGGCCGC**CAGTTGTAGATTCAGAGAATAG |
| 1726 | 5’ trunc *gerS* SOE to *CD3465* | CTGAAGTAGGAGGTGTGATGTGCAAAAACGACAGTCCAC |
| 1727 | 3’ *CD3465* SOE to trunc *gerS* | GTGGACTGTCGTTTTTGCACATCACACCTCCTACTTCAG |
| 1733 | 5’ C22S *gerS* SOE | GCACTGGTTATAATTGTAATAGGATCCCAAAAACGACAGTCCACAAAAG |
| 1734 | 3’ C22S *gerS* rev oes | CTTTTGTGGACTGTCGTTTTTGGGATCCTATTACAATTATAACCAGTGC |
|  |  |  |
