# Supplementary material for: Identification of a Novel Lipoprotein Regulator of Clostridium difficile Spore Germination
Source: PLoS Pathog. 2015 Oct 23;11(10):e1005239. doi: 10.1371/journal.ppat.1005239 (PMC4619724; doi:10.1371/journal.ppat.1005239)
Supplement: S2 Table — (DOCX) [file ppat.1005239.s010.docx]

**Table S2. Raw data from heat-resistant spore formation assays**

*H.R. data shown in Figure 1*

|  |  |  | **Rep. 1** | **Rep. 2** | **Rep. 3** | **Rep. 1** | **Rep. 2** | **Rep. 3** |  |  |  |
| --- | --- | --- | --- | --- | --- | --- | --- | --- | --- | --- | --- |
| **Strain #** | **Strain** | **Condition** | **Cell Count** | **Cell Count** | **Cell Count** | **spore ratio** | **spore ratio** | **spore ratio** | **Avg.** | **std. dev** | **Eff.** |
| 11 | JIR8094 | No Heat | 6,000,000 | 11,333,303 | 10,000,000 | 0.008 | 0.006 | 0.007 | 0.007 | 0.001 | 1.0 |
| 35 | *spo0A-* | No Heat | 7,333,330 | 13,333,303 | 9,333,330 | – | – | – | – | – | – |
| 278 | *gerS^–^* | No Heat | 10,000,000 | 7,333,330 | 7,000,000 | – | – | – | – | – | – |
| 425 | *alr2*^–^ | No Heat | 10,000,000 | 8,000,000 | 8,000,000 | 0.02 | 0.01 | 0.05 | 0.027 | 0.021 | 3.8 |
| 11 | JIR8094 | Heat | 50,000 | 70,000 | 66,667 |  |  |  |  |  |  |
| 35 | *spo0A-* | Heat | – | – | – |  |  |  |  |  |  |
| 278 | *gerS^–^* | Heat | – | – | – |  |  |  |  |  |  |
| 425 | *alr2*^–^ | Heat | 200,000 | 80,000 | 400,000 |  |  |  |  |  |  |

*H.R. data shown in Figure S4*

|  |  |  | **Rep. 1** | **Rep. 2** | **Rep. 3** | **Rep. 1** | **Rep. 2** | **Rep. 3** |  |  |  |
| --- | --- | --- | --- | --- | --- | --- | --- | --- | --- | --- | --- |
| **Strain #** | **Strain** | **Condition** | **Cell Count** | **Cell Count** | **Cell Count** | **spore ratio** | **spore ratio** | **spore ratio** | **Avg.** | **std. dev** | **Eff.** |
| 111 | JIR8094/EV | No Heat | 17,666,667 | 18,000,000 | 14,000,000 | 0.077 | 0.046 | 0.031 | 0.052 | 0.024 | 1.0 |
| 330 | *gerS^–^/EV* | No Heat | 26,666,667 | 8,000,000 | 15,000,000 | – | – | – | – | – | – |
| 521 | *gerS*^–^/*gerS* | No Heat | 14,333,333 | 17,666,667 | 12,666,667 | 0.074 | 0.049 | 0.047 | 0.057 | 0.015 | 1.1 |
| 628 | *gerS*^–^/single | No Heat | 10,333,333 | 25,333,333 | 10,666,667 | 0.061 | 0.017 | 0.044 | 0.041 | 0.022 | 0.8 |
| 111 | JIR8094/EV | Heat | 1,366,667 | 833,333 | 433,333 |  |  |  |  |  |  |
| 330 | *gerS^–^/EV* | Heat | – | – | – |  |  |  |  |  |  |
| 521 | *gerS*^–^/*gerS* | Heat | 1,066,667 | 866,667 | 600,000 |  |  |  |  |  |  |
| 628 | *gerS*^–^/single | Heat | 633,333 | 433,333 | 466,667 |  |  |  |  |  |  |

*H.R. data shown in Figure 7*

|  |  |  | **Rep. 1** | **Rep. 2** | **Rep. 3** | **Rep. 4** | **Rep. 5** | **Rep. 1** | **Rep. 2** | **Rep. 3** | **Rep. 4** | **Rep. 5** |  |  |
| --- | --- | --- | --- | --- | --- | --- | --- | --- | --- | --- | --- | --- | --- | --- |
| **Strain #** | **Sample** | **Condition** | **Cell Count** | **Cell Count** | **Cell Count** | **Cell Count** | **Cell Count** | **spore ratio** | **spore ratio** | **spore ratio** | **spore ratio** | **spore ratio** | **Avg.** | **std. dev** |
| 111 | JIR8094/EV | No Heat | 17,333,333 | 43,333,333 | 18,333,333 | 26,666,667 | 9,333,333 | 0.012 | 0.002 | 0.006 | 0.021 | 0.036 | 0.015 | 0.013 |
| 330 | *gerS–/EV* | No Heat | 9,666,667 | 11,000,000 | 14,666,667 | 40,000,000 | 5,666,667 | - | - | - | - | - | - | - |
| 521 | *gerS*^–^/*gerS* | No Heat | 16,000,000 | 4,000,000 | 30,000,000 | 13,000,000 | 9,333,333 | 0.023 | 0.004 | 0.007 | 0.006 | 0.029 | 0.014 | 0.011 |
| 641 | *gerS*^–^/ΔSP | No Heat | 16,000,000 | 9,666,667 | 20,000,000 | 33,333,333 | 8,666,667 | 0.015 | 0.003 | 0.015 | 0.007 | 0.123 | 0.033 | 0.051 |
| 630 | *gerS*^–^/C22S | No Heat | 20,000,000 | 8,333,333 | 10,666,667 | 18,000,000 | 8,333,333 | - | - | 1.6x10^-6^ | 1.1x10^-6^ | 2.4x10^-6^ | 1.0x10^-6^ | 1.0x10^-6^ |
| 111 | JIR8094/EV | Heat | 200,000 | 106,667 | 106,667 | 566,667 | 333,333 |  |  |  |  |  |  |  |
| 330 | *gerS–/EV* | Heat | - | - | - | - | - |  |  |  |  |  |  |  |
| 521 | *gerS*^–^/*gerS* | Heat | 366,667 | 14,333 | 200,000 | 76,667 | 266,667 |  |  |  |  |  |  |  |
| 641 | *gerS*^–^/ΔSP | Heat | 233,333 | 33,333 | 300,000 | 233,333 | 1,066,667 |  |  |  |  |  |  |  |
| 630 | *gerS*^–^/C22S | Heat | - | - | 17 | 20 | 20 |  |  |  |  |  |  |  |

Spore ratio is determined by dividing the number of CFUs obtained from the heat-treated sample relative to the untreated (“no heat”) sample for the same strain. Spore ratios were averaged. Rep. refers to the replicate number.
